# Pinoresinol Diglucoside Attenuates Nuclear Receptor Coactivator 4‐Mediated Ferritinophagy Associated with Cisplatin‐Induced Hearing Loss

**DOI:** 10.1002/advs.202408777

**Published:** 2025-03-07

**Authors:** Yin Chen, Cheng Cheng, Ao Li, Dengbin Ma, Siyu Li, Handong Wang, Song Gao, Dingding Liu, Panpan Song, Chenjie Yu, Xiaoyun Qian, Guoqiang Wan, Xia Gao

**Affiliations:** ^1^ Department of Otolaryngology Head and Neck Surgery Affiliated Drum Tower Hospital Medical School of Nanjing University No. 321 Zhongshan Road Nanjing 210008 China; ^2^ Research Institute of Otolaryngology No. 321 Zhongshan Road Nanjing Jiangsu 210061 China; ^3^ MOE Key Laboratory of Model Animal for Disease Study Department of Otolaryngology Head and Neck Surgery Jiangsu Provincial Key Medical Discipline (Laboratory) The Affiliated Drum Tower Hospital of Medical School Model Animal Research Center of Medical School Nanjing University Nanjing Jiangsu 210061 China

**Keywords:** cisplatin, ferritinophagy, ototoxicity, pinoresinol diglucoside, Socs1

## Abstract

Cisplatin can cause irreversible hearing loss. However, effective approaches to its prevention are not established. In this study, the effect of the traditional Chinese medicine monomer pinoresinol diglucoside (PDG) is evaluated on cisplatin‐induced ototoxicity and its underlying mechanism of action. PDG significantly increases cell viability and inhibits reactive oxygen species production and ferroptosis in cisplatin‐treated House Ear Institute‐Organ of Corti 1 cells and basilar membranes. PDG partially restores hearing loss caused by cisplatin. Transcriptome sequencing identifies Suppressor of Cytokine Signaling 1 (SOCS1), which is significantly elevated in the cisplatin‐only group but significantly reduced after PDG application. SOCS1 is a ferroptosis‐promoting factor, and knocking it down significantly inhibits nuclear receptor coactivator 4 (NCOA4) and inhibits ferritinophagy. Transmission electron microscopy reveals that knocking down SOCS1 reduces the number of autophagic lysosomes induced by cisplatin. Co‐immunoprecipitation is performed to confirm the interaction between SOCS1 and NCOA4. Taken together, these results indicate that PDG inhibits NCOA4‐mediated ferritinophagy by downregulating SOCS1, which reduces cisplatin‐induced ototoxicity. This study provides a new clinical option for the prevention of cisplatin‐induced hearing loss.

## Graphical abstract

Pinoresinol diglucoside, a bioactive component extracted from traditional Chinese medicine, attenuates cisplatin‐induced ototoxicity in vivo and in vitro. The protective effect is achieved by downregulation of *Socs1*. The present study is the first to investigate the expression and function of *Socs1* in inner ear, which is known to promote ferroptosis. Furthermore, downregulating *Socs1* can inhibit NCOA4‐mediated ferroautophagy.

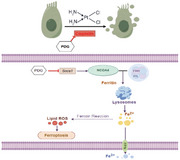

## 1 Introduction

Cisplatin is widely recognized among the most potent anticancer medications due to its remarkable therapeutic efficacy against various cancer types.^[^ ^1^ ^]^ Nonetheless, repeated administration of cisplatin can result in ototoxicity, with an incidence ranging from 40% to 80%.^[^ ^2^ ^]^ Cisplatin disrupts the homeostasis of the inner ear lymph. This subsequently induces persistent oxidative stress through the production of reactive oxygen species (ROS) and triggers multiple cell death.^[^ ^3^ ^]^ Mammalian hair cells (HCs) cannot be regenerated once lost, and cisplatin‐induced sensorineural hearing loss is irreversible. No curative treatments are currently available.^[^ ^4^ ^]^

In the realm of Chinese herbal medicine, there is a growing interest in leveraging ancient wisdom to combat modern medical challenges, such as cisplatin‐induced ototoxicity.^[^ ^5^, ^6^ ^]^ Pinoresinol diglucoside (PDG) is a bioactive component that can be extracted from traditional Chinese medicines such as Eucommia ulmoides, Styrax sp., and Forsythia suspense.^[^ ^7^ ^]^ As a bicyclic epoxy lignan, PDG exhibits diverse pharmacological properties such as reduced oxidative stress and inflammation. Studies have shown that PDG can suppress the generation of ROS and malondialdehyde (MDA) by inhibiting several inflammation‐associated pathways such as MAPK and NF‐kB both in vitro and in vivo.^[^ ^7^, ^8^, ^9^, ^10^ ^]^

Regulated cell death, particularly ferroptosis, an iron‐dependent regulated cell death characterized by lipid peroxidation,^[^ ^11^ ^]^ is increasingly recognized as a significant player in the pathogenesis of cisplatin‐induced ototoxicity. Cisplatin promotes ototoxicity by inducing ferroptosis, and there is evidence that inhibiting ferroptosis can alleviate cisplatin‐induced hearing loss.^[^ ^12^ ^,‐^ ^14^ ^]^ Iron accumulation is critical for ferroptosis and inhibited nuclear receptor coactivator 4 (NCOA4)‐mediated ferritinophagy is necessary for the induction of ferroptosis. Ferritinophagy is a process of selective autophagy facilitated by NCOA4, which involves the identification of ferritin by NCOA4 and its subsequent transport to the autophagosome.^[^ ^15^, ^16^, ^17^ ^]^ Our study investigates whether PDG can mitigate cisplatin‐induced hearing loss by inhibiting NCOA4‐mediated ferritinophagy.

This research aims to not only indicate the effects of PDG against cisplatin‐induced ototoxicity but also to explore its underlying mechanisms, potentially paving the way for new therapeutic strategies of traditional Chinese medicine components in modern clinical settings.

## 2 Results

### 2.1 PDG Attenuates Oxidative Stress Caused by Cisplatin in House Ear Institute‐Organ of Corti 1 Cells

**Figure 1:**
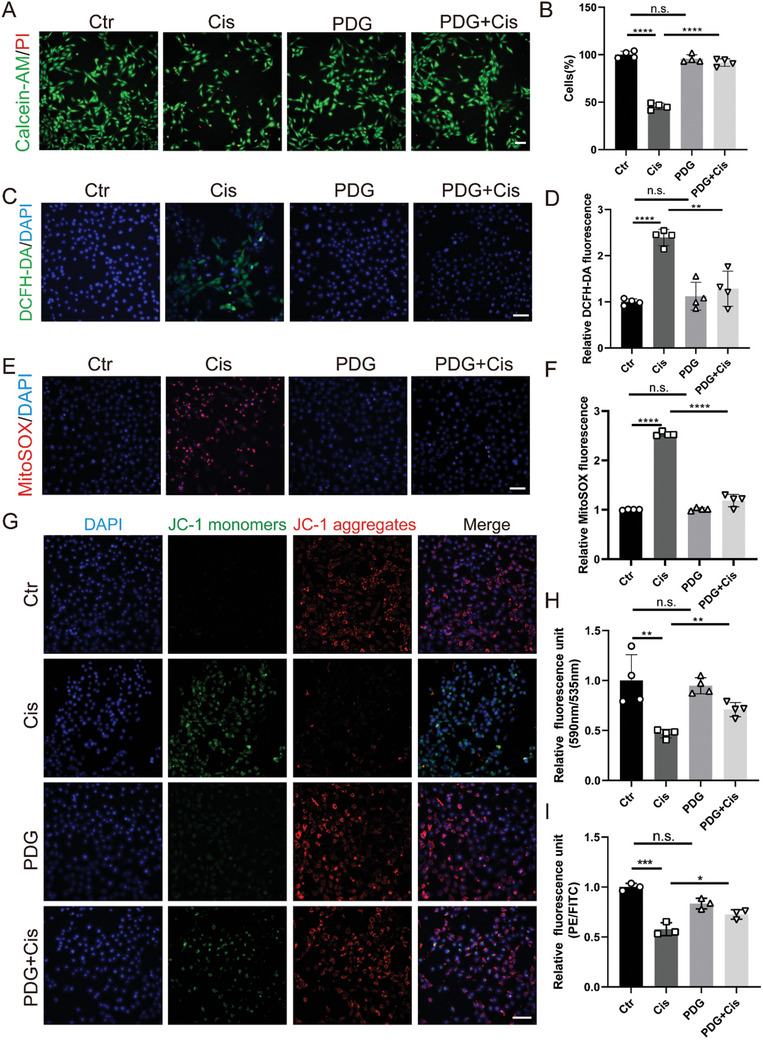
PDG alleviates cisplatin‐induced oxidative stress in HEI‐OC1 cells. A) Live (green)/dead (red) staining images of HEI‐OC1 cells incubated under different treatments; *n* = 4. Scale bar: 100 µm. B) Quantitative analysis of the relative fluorescence intensity of Live/Dead staining; *n* = 4. C) DCFH‐DA labeled with green fluorescence; *n* = 4. Scale bar: 100 µm. D) Quantitative analysis of the relative fluorescence intensity of DCFH‐DA; *n* = 4. E) MitoSOX labeled with red fluorescence; *n* = 4. Scale bar: 100 µm. F) Quantitative analysis of the relative fluorescence intensity of MitoSOX; *n* = 4. G) Mitochondrial membrane potential labeled with JC‐1 probe; *n* = 4. Scale bar: 100 µm. H) Quantitative analysis of the relative fluorescence intensity of JC‐1; *n* = 4. I) Quantitative analysis of the relative fluorescence intensity of JC‐1 in different groups via flow cytometry; *n* = 3. The data are presented as mean ± SD. *p < 0.05, **p < 0.01, ***p < 0.001, ****p < 0.0001, n.s. = not significant, two‐tailed, unpaired Student's *t*‐tests.

House Ear Institute‐Organ of Corti 1 (HEI‐OC1) cells were used to evaluate the protective efficacy of PDG against cisplatin‐induced HC damage. We tested the effect of various cisplatin concentrations on cell viability. The optimal concentration was 30 µm for 24 h resulting in ≈50% cell viability (Figure S1A, Supporting Information). We also determined whether PDG had cytotoxicity and found that concentrations as high as 200 µm did not have adverse effects on the viability of HEI‐OC1 cells (Figure S1B, Supporting Information). Co‐culturing 30 µm of cisplatin with varying concentrations of PDG (1, 5, 10, 25, 50, and 100 µm) for 24 h showed that PDG had the greatest protective effect at 25 µm. This resulted in 92.6% cell survival after cisplatin treatment (Figure S1C, Supporting Information). Subsequent cell experiments were carried out using 25 µm of PDG and 30 µm of cisplatin. Cell viability was confirmed by dual staining with calcein‐AM (green) and propidium iodide (red) (**Figure** **1**A). Quantitative analysis revealed a cell survival rate of 45.6% in the cisplatin group, whereas the PDG and PDG+Cisplatin groups had survival rates of 95.8% and 91.7%, respectively (Figure 1B). We also used the Cal‐27 cell line (a tongue cancer cell line) to verify that the application of PDG did not compromise the antitumor activity of cisplatin. Our results further substantiated the preservation of the antitumor efficacy of cisplatin in the presence of PDG (Supplementary Figure S1D–F, Supporting Information).

Excessive ROS production is the primary contributor to cisplatin‐associated cell death,^[^ ^18^ ^]^ and mitochondria are among the principal sites of ROS production. The ROS levels in cisplatin‐treated HEI‐OC1 cells were evaluated using DCFH‐DA, MitoSOX, and JC‐1. DCFH‐DA was used to detect the intracellular ROS levels. MitoSOX is a fluorescent probe for the detection of superoxide in mitochondria. The increased production of ROS within the mitochondria was evidenced by an increase in JC‐1 monomers as the mitochondrial membrane potential decreased. As shown in our results, the cisplatin group had more DCFH‐DA‐ and MitoSOX‐positive cells and a lower mitochondrial membrane potential than the control group, indicating increased ROS production following cisplatin treatment. Conversely, DCFH‐DA, MitoSOX, and JC‐1 monomers were markedly reduced in the PDG+Cisplatin group relative to the cisplatin group. This suggested that PDG may mitigate excessive ROS accumulation. Moreover, PDG alone did not affect the ROS levels relative to the control group (Figure 1C–I).

### 2.2 PDG Inhibits Cisplatin‐Induced Ferroptosis in HEI‐OC1 Cells

The accumulation of lipid peroxides and the deposition of free iron are key factors in ferroptosis, and we analyzed cellular lipid peroxides using a Lipid Peroxidation Malondialdehyde (MDA) Assay Kit and Lipid Peroxidation Probe BDP 581/591 C11. Some fatty acids, such as MDA, can be oxidized and decomposed into complex compounds. Thus, MDA is commonly used as an indicator of lipid oxidation. We also assessed mitochondrial free iron ions with Mito‐FerroGreen and cellular free iron ions with the Ferro‐Orange and Iron Assay Kit to monitor the onset of ferroptosis and the potential inhibitory effects of PDG on cisplatin‐induced ferroptosis.

**Figure 2:**
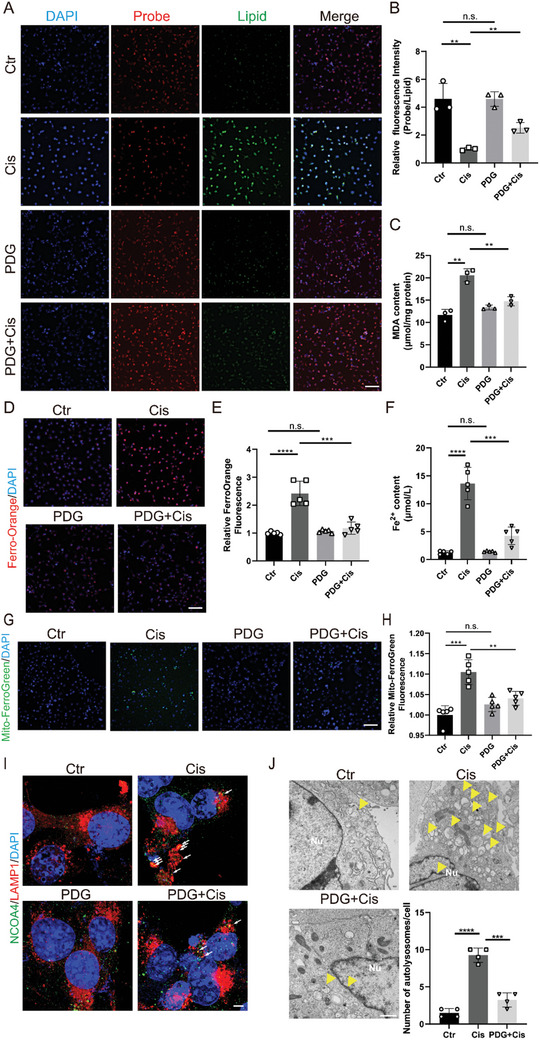
PDG alleviates cisplatin‐induced ferroptosis in HEI‐OC1 cells. A) Peroxidation products were labeled with the BDP probe; *n* = 3. Scale bar: 100 µm. B) Quantitative analysis of the relative fluorescence intensity of BDP; *n* = 3. C) MDA concentrations in HEI‐OC1 cells after different treatments; *n* = 3. D) Ferrous ions in the cell labeled with Ferro‐Orange; *n* = 5. Scale bar: 100 µm. E) Quantitative analysis of the relative fluorescence intensity of Ferro‐Orange; *n* = 5. F) The content of Fe^2+^ in HEI‐OC1 cells in different groups; *n* = 5. G) Ferrous ions in the mitochondria labeled with Mito‐FerroGreen; *n* = 5. Scale bar: 100 µm. H) Quantitative analysis of the relative fluorescence intensity of Mito‐FerroGreen; *n* = 5. I) Immunofluorescence images showed co‐localization of NCOA4 and LAMP1 in HEI‐OC1 cells. White arrows indicate the co‐localization; *n* = 4. Scale bar = 5 µm. J) Representative transmission electron microscopy images of autolysosomes in HEI‐OC1 cells following different treatments. Yellow arrows indicate autolysosomes; *n* = 3. Scale bar: 1 µm. The data are presented as mean ± SD. *p < 0.05, **p < 0.01, ***p < 0.001, ****p < 0.0001, n.s. = not significant, two‐tailed, unpaired Student's *t*‐tests.

BDP staining yielded comparable results, with minimal lipid peroxide production in the control group, substantial lipid peroxide production in the cisplatin group, and a significant decrease in cisplatin‐induced lipid peroxide accumulation in the PDG+Cisplatin group (**Figure** **2**A,B). Similarly, the cisplatin group showed a two‐fold increase in MDA production, while the PDG+Cisplatin group showed results similar to those of the control group. In contrast, cells in the PDG+Cisplatin group exhibited a notable decrease in MDA production relative to the cisplatin group (Figure 2C). These results suggest that cisplatin can lead to marked lipid peroxide production, and PDG can reduce the cisplatin‐induced accumulation of these lipid peroxides.

Ferro‐orange staining showed a 1.5‐fold increase in fluorescence intensity following treatment with cisplatin and a decrease to 73.3% in the PDG+Cisplatin group relative to the cisplatin group (Figure 2D,E). The quantitative statistics for Fe2+ in HEI‐OC1 cells showed similar results (Figure 2F). These findings suggest that PDG can effectively mitigate cisplatin‐induced accumulation of intracellular iron ions. Similarly, staining with Mito‐FerroGreen revealed the presence of iron ions in the mitochondria (Figure 2G). Quantitative analysis indicated that the relative fluorescence intensity of the cisplatin group was 1.1 times higher than that of the control group, whereas that of the PDG+Cisplatin group was significantly lower than that of the cisplatin group (Figure 2H).

NCOA4‐mediated ferritinophagy is required for the induction of ferroptosis, and we further investigated the effects of ferritinophagy in HEI‐OC1 cells. We assessed the combination of NCOA4 and LAMP1 (a known lysosomal marker) using immunostaining. Our results revealed that the co‐localization of NCOA4 and LAMP1 was significantly increased in the cisplatin‐treated group, indicating that cisplatin‐induced ferritinophagy. The co‐localization decreased after PDG treatment, suggesting that PDG could inhibit NCOA4‐mediated ferroautophagy (Figure 2I). Transmission electron microscopy revealed that PDG significantly reduced cisplatin‐induced autophagolysosome production in HEI‐OC1 cells (Figure 2J). These results suggested that NCOA4‐induced ferritinophagy caused by cisplatin could be decreased by PDG in HEI‐OC1 cells. Our results demonstrate the ability of PDG to inhibit cisplatin‐induced ferroptosis and ferritinophagy in HEI‐OC1 cells.

### 2.3 PDG Inhibits Cisplatin‐Induced Ototoxicity and Ferroptosis In Vivo and In Vitro

**Figure 3:**
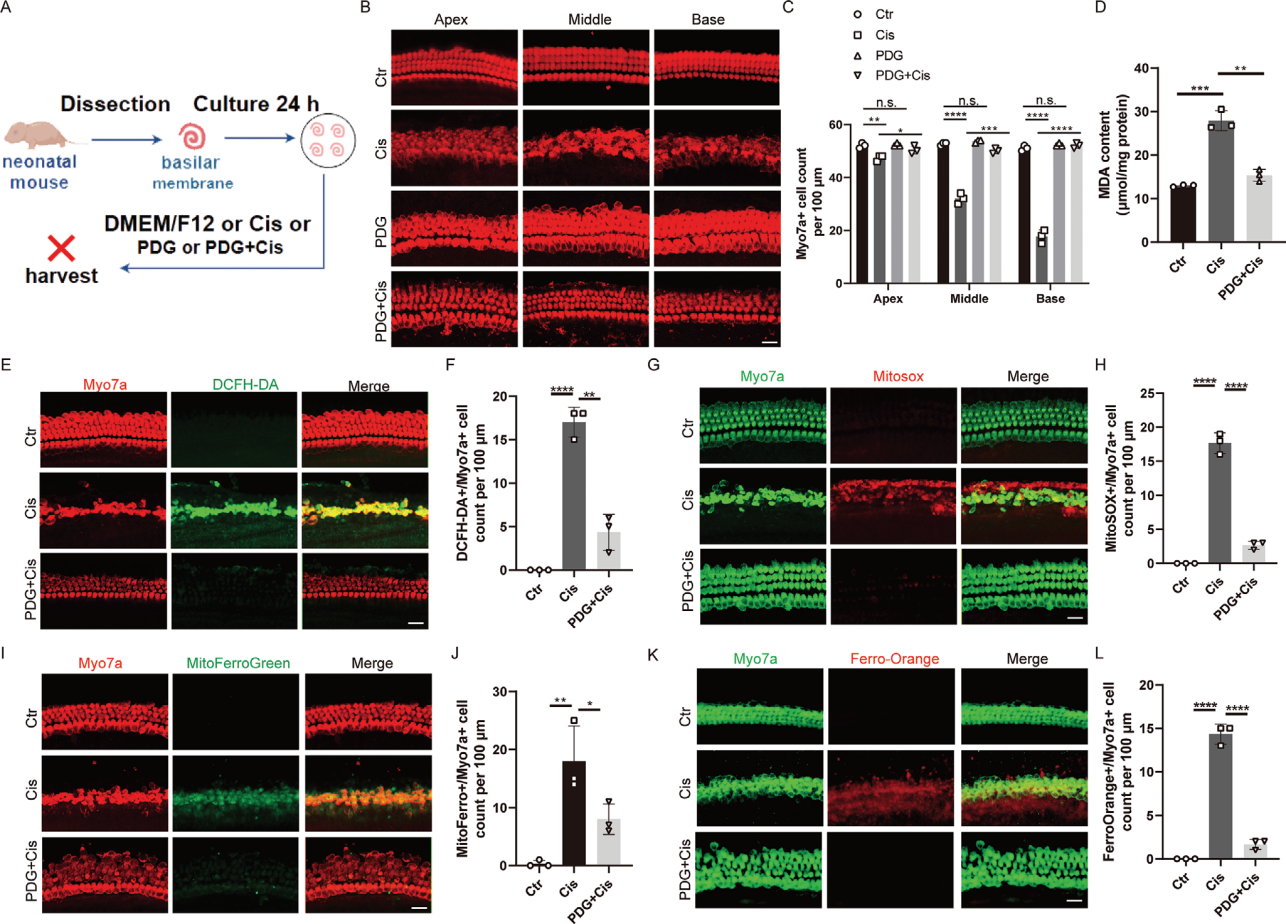
PDG alleviates cisplatin‐induced oxidative stress and ferroptosis in ex vivo cultures. A) Experimental protocol for PDG protection against cisplatin‐induced ototoxicity. B) Representative images of basilar membranes labeled with MyosinVIIa (red) from the different groups; *n* = 3. Scale bar: 20 µm. C) Quantitation of MyosinVIIa‐positive HCs in different regions of basilar membranes in various groups; *n* = 3. D) MDA concentrations in basilar membranes after different treatments; *n* = 3. E) Immunostaining of DCFH‐DA (green) in the basal turn of basilar membranes from the different groups. HCs are labeled with MyosinVIIa (red); *n* = 3. Scale bar: 20 µm. F) Quantitation of MyosinVIIa and DCFH‐DA double‐positive HCs in the basal turn of basilar membranes from various groups; *n* = 3. G) Images of MitoSOX staining (red) in the basal turn of basilar membranes from the different groups. HCs are labeled with MyosinVIIa (green); *n* = 3. Scale bar: 20 µm. H) Quantitation of MyosinVIIa and MitoSOX double‐positive HCs in the basal turn of basilar membranes from the various groups; *n* = 3. I) Images of Mito‐FerroGreen in the basal turn of the basilar membranes from different groups. HCs are labeled with MyosinVIIa (red); *n* = 3. Scale bar: 20 µm. J) Quantitation of MyosinVIIa and Mito‐FerroGreen double‐positive HCs in the basal turn of the basilar membranes for various groups; *n* = 3. K) Images of Ferro‐Orange in the basal turn of the basilar membranes from different groups. HCs are labeled with MyosinVIIa (green); *n* = 3. Scale bar: 20 µm. L) Quantitation of MyosinVIIa and Ferro‐Orange double‐positive HCs in the basal turn of the basilar membranes for various groups; *n* = 3. The data are presented as mean ± SD. *p < 0.05, **p < 0.01, ***p < 0.001, ****p < 0.0001, n.s. = not significant, two‐tailed, unpaired Student's *t*‐tests.

To determine whether PDG can protect HCs against cisplatin‐induced injury in vitro, basilar membranes from neonatal wild‐type C57BL/6 mice (at postnatal day (P) 3) were treated with 30 µm cisplatin and different concentrations of PDG (10, 25, 50, and 100 µm) (Figure S2A, Supporting Information). HCs were stained with MyosinVIIa antibody and were markedly decreased in the cisplatin group. Treatment with 25 µm PDG led to a marked increase in the amount of HCs following cisplatin exposure (Figure S2B, Supporting Information). Therefore, 25 µm was determined to be the optimal concentration of PDG for subsequent experiments involving basilar membranes (**Figure** **3**A). HC loss in all three regions of the cochlea (base, middle, and apex) was significantly increased in the cisplatin group. In contrast, treatment with PDG significantly attenuated cisplatin‐induced HC loss (Figure 3B,C). The cisplatin group, relative to the control group, showed a 2.2‐fold increase in MDA concentrations in the basilar membranes. The PDG+Cisplatin group, relative to the cisplatin group, showed a 0.45‐fold decrease in the MDA concentrations (Figure 3D). Immunofluorescence labeling with DCFH‐DA and MitoSOX indicated higher ROS levels in the cisplatin group (Figure 3E–H). PDG treatment in the PDG+Cisplatin group resulted in a decrease in DCFH‐DA‐ and MitoSOX‐positive HCs, suggesting that PDG can reduce cisplatin‐induced ROS accumulation. In addition, the cisplatin‐treated basilar membranes had more Mito‐FerroGreen‐ and Ferro‐Orange‐positive HCs than the control group, and the number of Mito‐FerroGreen‐ and Ferro‐Orange‐positive HCs in the PDG+Cisplatin group was remarkably reduced (Figure 3I–L). This confirmed previous results in the HEI‐OC1 cells showing that PDG can mitigate cisplatin‐induced iron accumulation in mitochondria. These findings indicate that PDG protects against cisplatin‐induced HC death by reducing ROS levels and inhibiting ferroptosis in vitro.

**Figure 4:**
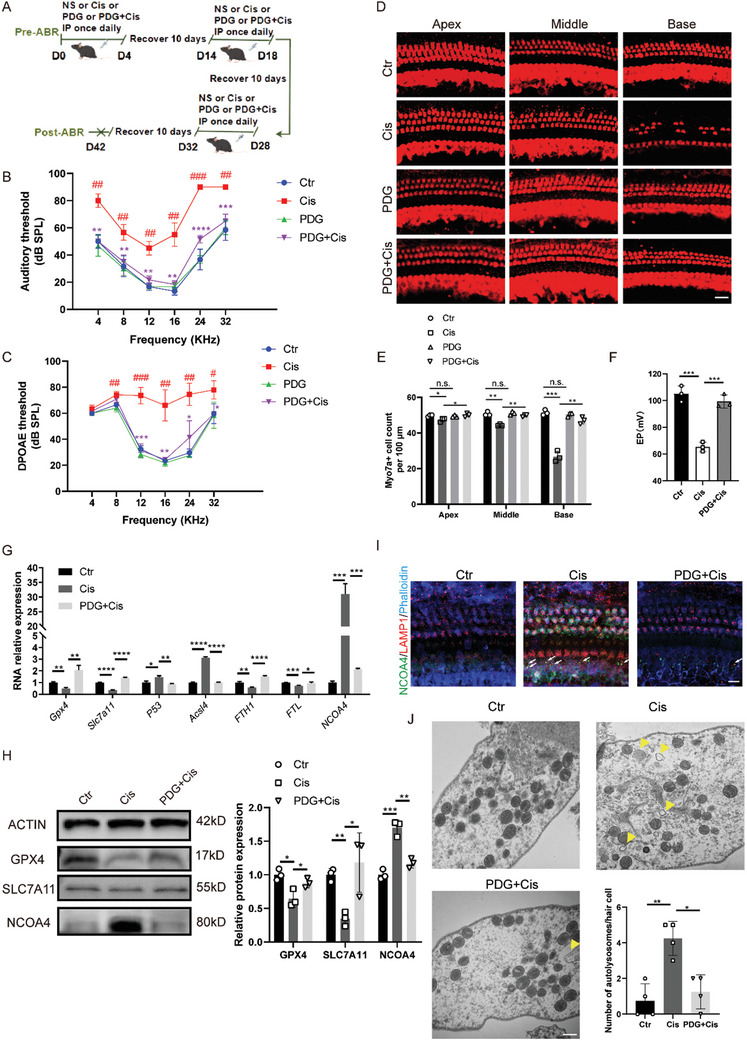
PDG protected HCs from cisplatin‐induced hearing loss in vivo. A) Experimental protocol for animal testing. B) ABR thresholds after different treatments; *n* = 5. C) Thresholds of DPOAE after different treatments; *n* = 5. D) Representative MyosinVIIa (red)‐stained confocal images of cochlear HCs from different groups; *n* = 3. Scale bar: 20 µm. E) The number of HCs per 100 µm in various cochlear regions was counted by MyosinVIIa staining; *n* = 3. F) EP values of endolymph potential recorded from the basal cochlear turns; *n* = 3. G) Quantitative polymerase chain reaction analysis of ferroptosis‐related genes in vivo with different treatments; *n* = 4. H) Western blotting analysis of FTH1, NCOA4, and GPX4 in different groups; *n* = 3. I) Immunofluorescence images showed co‐localization of NCOA4 and LAMP1. White arrows indicate co‐localization; *n* = 3. Scale bar = 10 µm. J) Representative transmission electron microscopy images of autolysosomes in hair cells following different treatments. Yellow arrows indicate autolysosomes; *n* = 3. Scale bar: 500 nm. The data are presented as mean ± SD. *p < 0.05, **p < 0.01, ***p < 0.001, ****p < 0.0001, n.s. = not significant versus the Cisplatin group and ##*p* < 0.01 and ###*p* < 0.001 versus the Control group, two‐tailed, unpaired Student's *t*‐tests.

To examine the effects of PDG in vivo, we used a previously documented cisplatin‐induced in vivo injury model ^[^ ^19^ ^]^ (**Figure** **4**A). Following various treatments, the auditory brainstem response (ABR) results indicated that the cisplatin group had higher hearing thresholds than the control group across all frequencies tested. A 25 mg kg^−1^ dose of PDG mitigated the cisplatin‐induced increase in the ABR threshold (Figure 4B; Figure S3, Supporting Information). Distortion product otoacoustic emission (DPOAE) was used to evaluate the function of cochlear outer HCs (Figure 4C). The DPOAE results showed that the threshold in the cisplatin group was significantly increased at 8, 12, 16, 24, and 32 kHz, which proved that cisplatin severely damaged the outer HC. However, the threshold of the PDG + Cisplatin group significantly decreased. Moreover, PDG alone had little effect on the DPOAE threshold. Immunofluorescence staining with anti‐MyosinVIIa antibody revealed a significant decrease in the number of HCs following cisplatin treatment and a notable increase in the number of surviving HCs, while PDG alone did not affect their survival (Figure 4D,E). The endocochlear potential (EP) is also an important parameter for verifying the protection of PDG in the cochlear electrochemical environment. Mice in the cisplatin group had >40% reduction in EP values relative to the control group, and the EP of the PDG+Cis group significantly increased (Figure 4F). Overall, these findings indicate that PDG prevents cisplatin‐induced HC loss in mice.

Furthermore, the expression levels of ferroptosis‐related markers, including Gpx4, Slc7a11, P53, and Ncoa4, were detected, and we found that PDG effectively inhibited cisplatin‐induced ferroptosis (Figure 4G,H). Furthermore, immunofluorescence was used to confirm the co‐localization of NCOA4 and LAMP1 to demonstrate that PDG could inhibit NCOA4‐induced ferritinophagy, an important mechanism of ferroptosis. Our results showed that the co‐localization of NCOA4 and LAMP1 significantly increased in the cisplatin group and decreased after PDG treatment (Figure 4I). Similarly, the TEM results showed that PDG reduced the increase in autophagic lysosomes in the cisplatin group (Figure 4J). These results indicated that PDG effectively inhibited ferroptosis in vivo, including NCOA4‐mediated ferritinophagy.

### 2.4 Inhibition of Cisplatin‐Induced Ototoxicity by PDG was Associated with a Decrease in SOCS1

**Figure 5:**
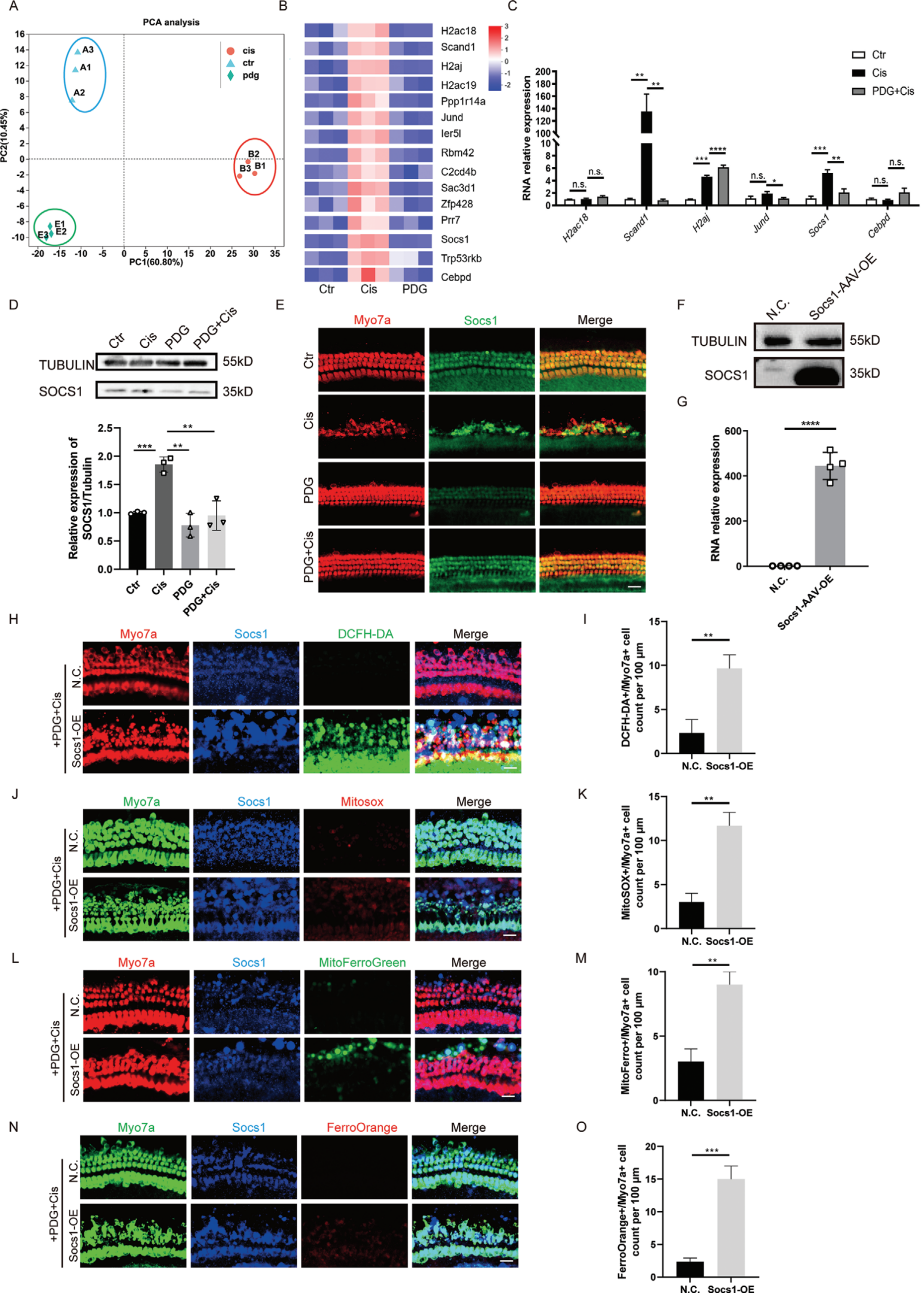
PDG reduced cisplatin‐induced HC death by downregulating SOCS1. A,B) PCA analysis A) and heatmap B) of differentially expressed genes in the Control (Ctr), Cisplatin (Cis), and PDG groups. C) Q‐PCR analysis of significantly different genes; *n* = 4. D) Relative expression levels of SOCS1 in different treatment groups determined by WB analysis; *n* = 3. E) Immunofluorescence staining for MyosinVIIa (red) and SOCS1 (green) showed SOCS1 expressions in different treatment groups; *n* = 3. Scale bar: 20 µm. Relative protein F) and RNA G) expressions of SOCS1 in basilar membranes transfected with SOCS1‐AAV‐OE; *n* = 4. H) Images of DCFH‐DA staining (green) in the basal turn of basilar membranes from the different groups. HCs are labeled with MyosinVIIa (red); *n* = 3. Scale bar: 20 µm. I) Quantitation of MyosinVIIa and DCFH‐DA double‐positive HCs in the basal turn of basilar membranes from various groups; *n* = 3. J) Images of MitoSOX staining (red) in the basal turn of basilar membranes from the different groups. HCs are labeled with MyosinVIIa (green); *n* = 3. Scale bar: 20 µm. K) Quantitation of MyosinVIIa and MitoSOX double‐positive HCs in the basal turn of basilar membranes from the various groups. *n* = 3. L) Images of Mito‐FerroGreen in the basal turn of the basilar membranes from different groups. HCs are labeled with MyosinVIIa (red); *n* = 3. Scale bar: 20 µm. M) Quantitation of MyosinVIIa and Mito‐FerroGreen double‐positive HCs in the basal turn of the basilar membranes for various groups; *n* = 3. N) Images of Ferro‐Orange in the basal turn of the basilar membranes from different groups. HCs are labeled with MyosinVIIa (green); *n* = 3. Scale bar: 20 µm. O) Quantitation of MyosinVIIa and Ferro‐Orange double‐positive HCs in the basal turn of the basilar membranes for various groups; *n* = 3. The data are presented as the mean ± SD. *p < 0.05, **p < 0.01, ***p < 0.001, ****p < 0.0001, n.s. = not significant, two‐tailed, unpaired Student's *t*‐tests.

To further investigate the mechanism by which PDG attenuates cisplatin ototoxicity, we conducted RNA sequencing of basilar membranes from three experimental groups: control, cisplatin‐treated (cisplatin), and PDG‐treated (PDG) (**Figure** **5**A). We identified the top 15 genes upregulated in the cisplatin and control groups and downregulated in the PDG and cisplatin groups for further validation (Figure 5B). Of the differentially expressed genes validated by quantitative polymerase chain reaction (Q‐PCR), we observed marked increments in the expressions of SCAND1 and SOCS1 in the cisplatin group, whereas their expressions declined in the PDG+Cisplatin group (Figure 5C). We also validated the expression of SOCS1 after different treatments in vivo and found that SOCS1 expression increased after cisplatin treatment and decreased after PDG treatment (Figure S4A–C, Supporting Information). Previous studies have demonstrated that SOCS1 is a regulator gene associated with ferroptosis and drives this process.^[^ ^20^, ^21^, ^22^, ^23^, ^24^ ^]^

Cochlear tissue was collected at P3, P7, P14, and P30 to examine the temporal and spatial expressions of SOCS1 in the mouse cochlea. Western blotting (WB) indicated that SOCS1 was expressed at all time points in the mouse cochlea, and the expression of SOCS1 did not change significantly over time (Figure S4D, Supporting Information). Immunofluorescence labeling using an anti‐MyosinVIIa antibody for HCs corroborated the WB findings, showing widespread expression of SOCS1 in the cochlear HCs (Figure S4E, Supporting Information). This demonstrated that the expression of SOCS1 in the mouse cochlea was not heterogeneous in time and space.

Furthermore, we performed a WB assay on the basilar membranes of the control, cisplatin, PDG, and PDG+Cisplatin groups. The results of the WB assay were consistent with the sequencing and Q‐PCR data. The expression of SOCS1 in the basilar membranes was significantly increased after treatment with cisplatin. However, the application of PDG alone significantly reduced the expression of SOCS1 (Figure 5D,E).

We utilized an adeno‐associated virus (AAV) to overexpress SOCS1 in the PDG+Cisplatin group to verify its function in the basilar membranes (Figure 5F,G). As shown in our results, the PDG+Cisplatin+SOCS1 overexpression (OE) group had more PI‐positive cells, DCFH‐DA‐positive cells, and MitoSOX‐positive cells than the PDG+Cisplatin group, indicating increased ROS production with the overexpression of SOCS1 (Figure S5A, Supporting Information). Furthermore, more Mito‐FerroGreen‐, Ferro‐Orange‐, and BDP‐lipid‐positive cells were observed in the PDG+Cisplatin+SOCS1 OE group, demonstrating that the overexpression of SOCS1 increased ferroptosis in HEI‐OC1 cells (Figure S5B, Supporting Information). Similarly, SOCS1 overexpression resulted reductions in DCFH‐DA, MitoSOX, Mito‐FerroGreen, and Ferro‐Orange‐positive HCs in the PDG+Cisplatin group relative to the PDG+Cisplatin group (Figure 5H–O). This confirmed previous results in HEI‐OC1 cells.

These findings suggested that PDG exerts its effects by downregulating the SOCS1 gene to inhibit ototoxicity.

### 2.5 Knockdown of SOCS1 Attenuates Ferroptosis in Basilar Membranes

**Figure 6:**
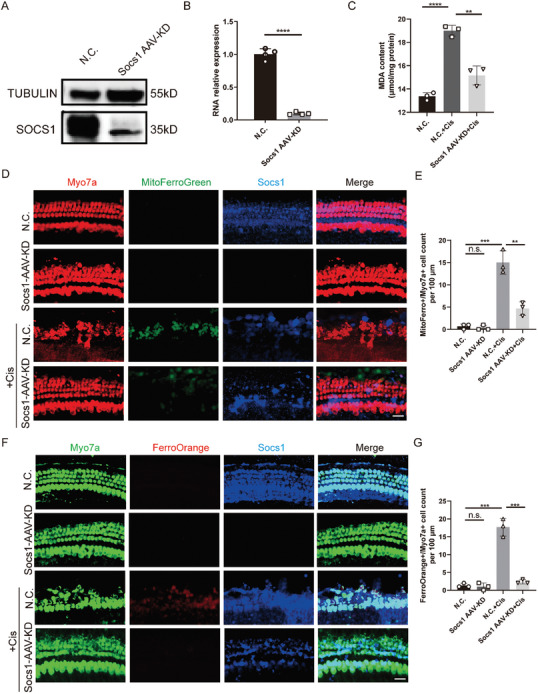
Downregulating SOCS1 alleviated NCOA4‐induced ferritinophagy in ex vivo cultures. A,B) Relative protein and RNA expressions of SOCS1 in basilar membranes after the downregulation of SOCS1 expression; *n* = 4. C) MDA concentrations in basilar membranes transfected with AAV‐SOCS1; *n* = 3. D) Images of Mito‐FerroGreen in the basal turn of basilar membranes after SOCS1 knockdown. HCs are labeled with MyosinVIIa (red); *n* = 3. Scale bar: 20 µm. E) Quantification of MyosinVIIa and Mito‐FerroGreen double‐positive HCs in the basal turn of the basilar membranes from the different groups; *n* = 3. F) Representative images of Ferro‐Orange in the basal turn of basilar membranes following different treatments after SOCS1 knockdown; *n* = 3. Scale bar: 20 µm. G) Quantification of MyosinVIIa and Ferro‐Orange double‐positive HCs in the basal turn of the basilar membranes from the different groups; *n* = 3. The data are presented as the mean ± SD. *p < 0.05, **p < 0.01, ***p < 0.001, ****p < 0.0001, n.s. = not significant, two‐tailed, unpaired Student's *t*‐tests.

We used AAV to knockdown SOCS1. This was to verify whether knocking down SOCS1 could inhibit ferroptosis in the basilar membranes. The efficacy of viral infection was confirmed through WB (**Figure** **6**A) and Q‐PCR (Figure 6B). The basilar membranes were exposed to cisplatin for 24 h at 72 h after AAV‐N.C. and AAV‐SOCS1‐KD infection. Knockdown of SOCS1 in cisplatin‐treated basilar membranes significantly reduced the production of MDA and Mito‐FerroGreen‐positive HCs and Ferro‐Orange‐positive HCs (Figure 6C–G). Overall, these results indicate that SOCS1 knockdown can inhibit cisplatin‐induced ferroptosis in HCs.

### 2.6 Downregulation of SOCS1 Contributes to Decreased of Ferritinophagy by Inhibited the Expression of NCOA4 In Vitro

**Figure 7:**
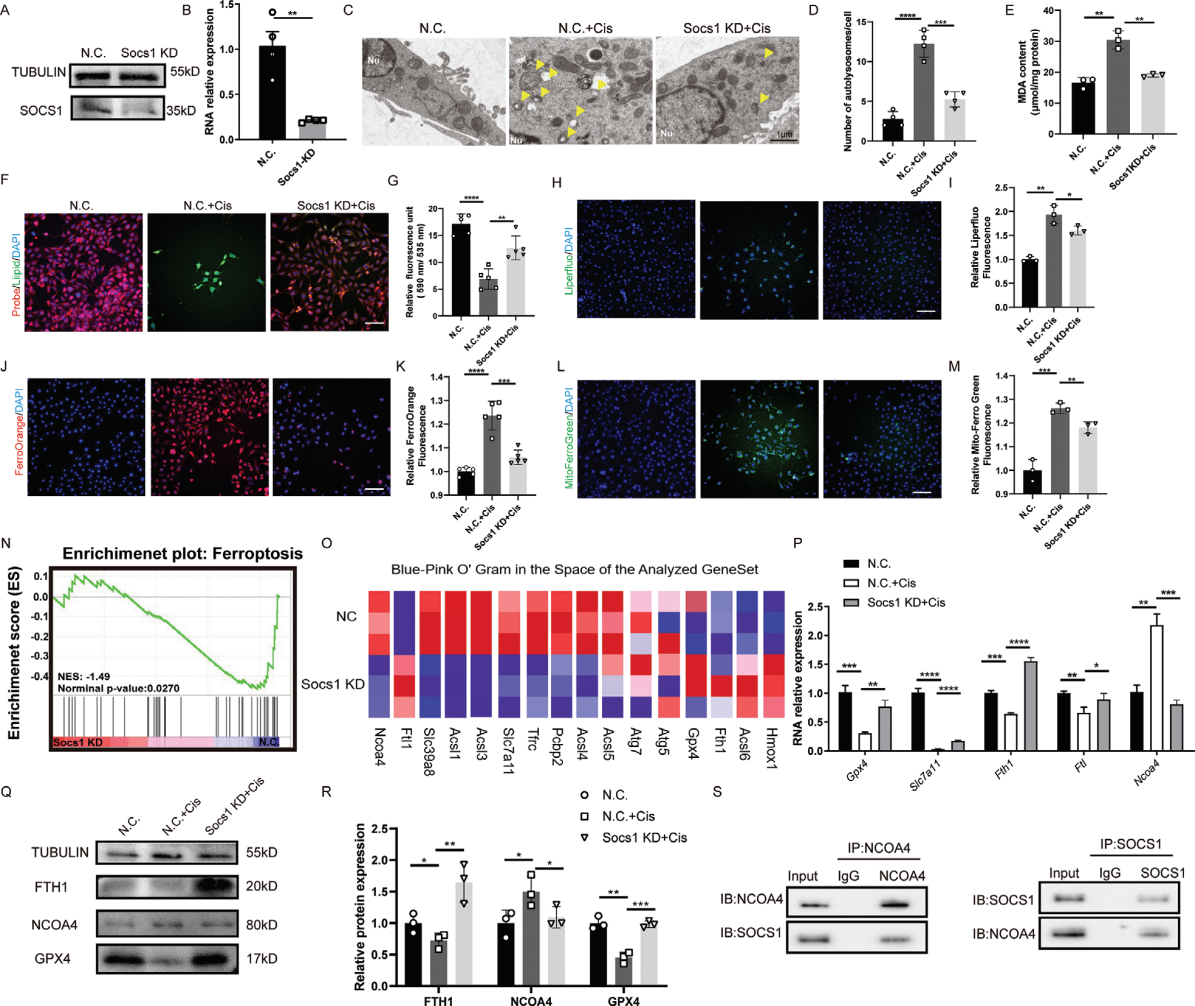
PDG alleviated NCOA4‐induced ferritinophagy by downregulating SOCS1 in HEI‐OC1 cells. Relative protein A) and RNA B) expressions of SOCS1 in HEI‐OC1 cells transfected with siRNA‐SOCS1; *n* = 4. C) MDA concentrations in cells transfected with siRNA‐SOCS1 after the different treatments; *n* = 3. D) Representative transmission electron microscopy images of autolysosomes in HEI‐OC1 cells following different treatments. Yellow arrows indicate autolysosomes; *n* = 3. Scale bar: 1 µm. E) Quantitative analysis of the autolysosomes per cell; *n* = 3. Lipid peroxidation labeled with BDP probe F) and Liperfluo H) in HEI‐OC1 cells with SOCS1 knockdown; *n* = 3. Scale bar: 50 µm. Quantitative analysis of the relative fluorescence intensity of BDP G) and Liperfluo I); *n* = 3. Ferrous ions in the cell labeled with Ferro‐Orange J) in HEI‐OC1 cells transfected with siRNA‐SOCS1. Ferrous ions in the mitochondria labeled with Mito‐FerroGreen L); *n* = 3. Scale bar: 100 µm. K) Quantitative analysis of the relative fluorescence intensity of Ferro‐Orange; *n* = 3. M) Quantitative analysis of the relative fluorescence intensity of Mito‐FerroGreen; *n* = 3. N) Gene Set Enrichment Analysis of differentially expressed genes in the N.C. group and the SOCS1 knockdown group. O) Blue‐Pink O’ gram of the analyzed gene set. P) Q‐PCR analysis of ferroptosis‐related genes in HEI‐OC1 cells with SOCS1 knockdown; *n* = 4. Q,R) WB analysis of FTH1, NCOA4, and GPX4 in HEI‐OC1 cells with SOCS1 knockdown; *n* = 3. S) Co‐immunoprecipitation analysis of the interaction between SOCS1 and NCOA4 in HEI‐OC1 cells; *n* = 3. The data are presented as the mean ± SD. *p < 0.05, **p < 0.01, ***p < 0.001, ****p < 0.0001, n.s. = not significant, two‐tailed, unpaired Student's *t*‐tests.

To validate these observations, we used small interfering RNA (siRNA) to silence SOCS1 in HEI‐OC1 cells. The transfection efficiency of siRNA‐SOCS1 was validated through WB (**Figure** **7**A) and Q‐PCR (Figure 7B), and SOCS1 KD was short for siRNA‐SOCS1 (Figure 7). The HEI‐OC1 cells were exposed to cisplatin for 24 h at 48 h after siRNA‐N.C. treatment and siRNA‐SOCS1 transfection. Further examination via transmission electron microscopy showed that knocking of SOCS1 can significantly reduce autophagolysosome production (Figure 7C,D). We found that knocking down SOCS1 in cisplatin‐treated cells significantly reduced MDA production (Figure 7E), decreased lipid peroxidation (as indicated by BDP staining), and reduced the number of Liperfluo‐positive cells (Figure 7F–I). These results suggest that knocking down SOCS1 can reduce the accumulation of lipid peroxidation in cells. Similarly, SOCS1 knockdown reduced the number of Ferro‐Orange‐positive cells and Mito‐FerroGreen‐positive cells (Figure 7J–M), indicating that knocking of SOCS1 can decrease free iron ions in cells and mitochondria. Overall, our results indicate that SOCS1 knockdown can inhibit cisplatin‐induced ferroptosis.

To further explore the changes in ferroptosis caused by knocking down SOCS1, we transfected cells with siRNA‐N.C. and siRNA‐SOCS1 for RNA sequencing. The results of gene set enrichment analysis (GSEA) indicated that ferroptosis was downregulated after knocking down SOCS1(Figure 7N), and we identified differentially expressed ferroptosis‐related genes (Figure 7O). Among these genes, the ferritinophagy‐related genes NCOA4, FTH1, and FTL1 showed significant changes. We verified the expressions of the ferroptosis markers GPX4 and SLC7A11 and the ferritinophagy‐related genes NCOA4, FTH1, and FTL1 using Q‐PCR. The results suggested that knocking down SOCS1 significantly increased the expressions of GPX4, SLC7A11, FTH1, and FTL but inhibited the expression of NCOA4 (Figure 7P). Subsequent validation by WB confirmed these Q‐PCR results, demonstrating elevated FTH1 and GPX4 expressions and decreased NCOA4 expression following SOCS1 knockdown (Figure 7Q,R). Additionally, Q‐PCR and WB in basilar membranes indicated the upregulation of the expression of FTH1 and downregulation of the expression of NCOA4 following SOCS1 knockdown (Figure S6, Supporting Information). These results suggest that knocking down SOCS1 in HEI‐OC1 cells and basilar membranes can similarly inhibit cisplatin‐induced ferritinophagy. Furthermore, our co‐immunoprecipitation analysis confirmed the interaction between SOCS1 and NCOA4 (Figure 7S), demonstrating that knocking down SOCS1 directly inhibits NCOA4‐mediated ferritinophagy.

## 3 Discussion

Inhibiting apoptosis only partially prevents cisplatin‐induced hearing loss.^[^ ^14^ ^]^ Ferroptosis is important in the development of cisplatin‐induced ototoxicity and is associated with increased lipid peroxidation and excessive generation of ROS due to the excessive accumulation of iron ions. Ferroptosis inhibitors can regulate ROS by inhibiting ferroptosis and protect HCs from damage.^[^ ^13^, ^14^, ^25^, ^26^ ^]^ In this study, we assessed ROS levels using DCFH‐DA and the mitochondria‐targeted ROS indicator MitoSOX Red; evaluated the mitochondrial membrane potential with JC‐1; detected cell lipid oxidation using Liperfluo, BDP, and MDA; and measured iron ion distribution using Ferro‐Orange and Mito‐FerroGreen. PDG treatment significantly reduced intracellular and mitochondrial ROS levels and prevented ferroptosis in HEI‐OC1 cells and HCs following cisplatin‐induced damage. Furthermore, the protective effect of PDG was preliminarily confirmed in spiral ganglion neuron (SGN) cells (Figure S7, Supporting Information), where PDG inhibited cisplatin‐induced ROS production and ferroptosis. Further specific protective mechanisms will be the target of our future investigations.

Ferritin is composed of ferritin light and heavy chain subunits (FTL and FTH1) and stores iron ions to maintain cellular iron homeostasis.^[^ ^27^, ^28^ ^]^ In the case of iron depletion, the ferritin‐specific autophagy adapter NCOA4 mediates macroautophagy to deliver ferritin to lysosomes, allowing the cells to use the stored iron.^[^ ^29^ ^]^ Autophagic degradation of excess ferritin has been shown to accumulate unstable iron and promote ferroptosis, which is called ferritinophagy. Inhibiting ferritinophagy by blocking autophagy or knocking down NCOA4 can reduce the accumulation of unstable iron and reactive oxygen species and inhibit ferroptosis in cells.^[^ ^17^, ^30^, ^31^ ^]^

Our results indicate that cisplatin activates ferritinophagy and leads to excessive release of free iron, lipid oxidation, and overproduction of ROS, resulting in HC death, which is consistent with previous findings.^[^ ^12^, ^26^, ^32^ ^]^ Furthermore, we validated the protective effects of PDG both in vitro and in vivo. In addition, the downregulation of SOCS1 contributed to the protective effect of PDG by inhibiting ferritinophagy.

Our transcriptome sequencing results showed that PDG can inhibit SOCS1 and reduce cisplatin‐induced hearing loss. Cytokines bind to specific receptors on the cell surface and transmit biological information to target cells to control and regulate fundamental biological processes, such as hematopoiesis, inflammation, immunity, and tumor development.^[^ ^33^ ^]^ SOCS proteins act as cytokine regulators and function as negative feedback inhibitors of cytokine‐induced signal transduction through the JAK/STAT pathway, with SOCS1 being the most effective member of the family capable of directly inhibiting JAK2.^[^ ^34^ ^]^ Recent studies have reported more applications for SOCS1 as a regulator of ferroptosis‐related genes. For example, wogonoside triggers ferroptosis through the SOCS1‐P53‐SLC7A11 pathway to alleviate liver fibrosis,^[^ ^20^ ^]^ and SOCS1 serves as a driver of ferroptosis by inhibiting the progression and chemoresistance of triple‐negative breast cancer.^[^ ^21^ ^]^ Furthermore, Mou et al. revealed that SOCS1 regulates iron metabolism and oxidative stress in macrophages by influencing transferrin receptor 1 and ferritin expression.^[^ ^35^ ^]^ However, the specific role of SOCS1 in promoting ferroptosis and its mechanism of action in cisplatin‐induced ototoxicity remain unknown.

We employed RNA transcriptome sequencing to compare cells with knocked‐down SOCS1 with negative control cells. Transcriptomic analysis revealed that SOCS1 plays an important role in ferroptosis. Gene set analysis revealed significant differences in ferritinophagy‐related genes, with NCOA4 emerging as key to ferritinophagy. Ferritin maintains cellular iron homeostasis by storing iron, and NCOA4 (the iron‐specific autophagy receptor) transports ferritin to lysosomes for iron ion release via macroautophagy.^[^ ^36^, ^37^ ^]^ We performed co‐immunoprecipitation for PDG‐targeted SOCS1 and NCOA4 in ferritinophagy, which indicated that SOCS1 and NCOA4 can interact. In addition, our in vitro experiments showed that cisplatin‐induced ferroptosis was significantly reduced after knocking down SOCS1, along with a decrease in the protein level of NCOA4. This further confirms the involvement of SOCS1 in PDG‐mediated HC protection. These findings confirmed that PDG regulates NCOA4‐mediated ferritinophagy by modulating SOCS1 to reduce cisplatin‐induced ototoxicity to provide a new avenue for future research.

Cisplatin can cause irreversible hearing damage in the inner ear by damaging hair cells, spiral neurons, and stria vascularis.^[^ ^2^, ^38^, ^39^, ^40^ ^]^ Our study fully demonstrated that PDG can effectively protect HCs in vivo and in vitro, and further explored the role of ferritinophagy in this process. However, there are several limitations to our study. While we have thoroughly investigated the protective effects of PDG on HCs, we did not further investigate the protective effect of PDG on SGN and stria vascularis. Specifically, we initially explored the protective effect of PDG in SGN cells, but did not further study the effect of PDG on SGN in vivo and the specific mechanism of how PDG affects SGN still needs to be further explored. Whether NCOA4‐mediated ferritinophagy also plays an important role in SGN is still unknown. Future research will be needed to explore these effects in greater detail and to better understand the potential therapeutic benefits of PDG for SGN protection.

In conclusion, this study showed that PDG treatment can prevent cisplatin‐induced ototoxicity in vitro and in vivo. The inhibition of excessive ROS and lipid peroxidation triggered by cisplatin, as well as the activation of the ferritinophagy pathway by PDG, is achieved through the inhibition of SOCS1. SOCS1 knockdown inhibits ferritinophagy and effectively alleviates cisplatin‐induced ototoxicity in vitro. A conditional knockout mouse model of SOCS1 in the cochlea was established, and we further validated the impact of SOCS1 knockout on cisplatin‐induced ferritinophagy in vivo. In summary, our current study suggests that PDG may be a potential drug for preventing cisplatin‐induced ototoxicity, and SOCS1 may be an important target for cisplatin‐induced ototoxicity.

## 4 Experimental Section

### Cell Culture and Drug Treatments

The HEI‐OC1 cells were cultured at 37 °C and 5% CO_2_, which was consistent with previous studies.^[^ ^41^, ^42^, ^43^ ^]^ HEI‐OC1 cells were exposed to cisplatin (B24462; Yuanye) at 30 µm for 24 h in the cisplatin group. In the PDG and PDG+Cisplatin groups, the cells were treated with PDG (HY‐N0657, MCE) for 24 h. Cisplatin was prepared using sterile phosphate‐buffered saline as a 3 mm solution, and PDG was prepared using DMSO as a 50 mm solution.

### Organ Culture of Basilar Membranes

Basilar membranes of C57BL6 mice at P3 were dissected in phosphate‐buffered saline (G4202; Servicebio) and affixed to coverslips coated with Cell‐Tak (354 240; Corning). The samples were cultured at 37 °C and 5% CO_2_. For the cisplatin toxicity test, the samples were exposed to 30 µm cisplatin for 24 h.

### Animal Models

Eight‐week‐old male C57BL/6 mice (purchased from Huachuang Sino) were used in this study and randomly assigned into four groups: control, cisplatin, PDG, and PDG+Cisplatin. A total of *n* = 10 mice per group were included to ensure statistical power. Mice in the cisplatin group were administered cisplatin injections for 42 days.^[^ ^44^ ^]^ Mice in the PDG+Cisplatin group received intraperitoneal injections of PDG (25 mg kg^−1^) concurrently with cisplatin. Mice in the control and PDG groups received injections of saline or PDG for four days. We prepared cisplatin as 0.5 mg mL^−1^ solution with sterile normal saline, and PDG as 2.5 mg mL^−1^ solution with 10% DMSO, 40% PEG300, and 5% Tween‐80. All experimental procedures adhered to the ethical guidelines for the treatment of laboratory animals and were approved by the Institutional Animal Care and Use Committee of Nanjing University (No. 2021AE01090). The study protocol was designed in compliance with the ARRIVE Guidelines 2.0 to ensure transparency and reproducibility (Supporting Information).

### Cell Viability Assay

Cell viability was assessed using the CCK‐8 kit (BMU106‐CN; Abbkine). The absorbance was measured at 450 nm using a microplate reader (Bio‐Rad Laboratories). The live/dead (Calcein‐AM/PI) double stain kit was used for a more direct evaluation of cytotoxicity. HEI‐OC1 cells were cultured as described above, and the kit was used according to the manufacturer's protocol to provide fluorescent images of live (green) and dead (red) cells. Each experiment was performed with five replicates.

### ROS Detection

DCFH‐DA (S0033M; Beyotime) was used to evaluate ROS levels, and MitoSOX Red (M36008; Invitrogen) was used to assess ROS levels in the mitochondria. HEI‐OC1 cells and basilar membranes were treated with DCFH‐DA or MitoSOX for 0.5 h in the cell incubator. HEI‐OC1 cells were visualized using a THUNDER imager (Leica) and examined on a BD Accuri C6 flow cytometer with FlowJo X software to quantify the results in the live state.

### Lipid Peroxidation Detection

Lipid peroxidation was assessed using two distinct methodologies: a colorimetric assay and a fluorescent probe technique. A Lipid Peroxidation MDA Assay Kit (S0131M, Beyotime) was used for the former, whereas the latter utilized the Lipid Peroxidation Probe BDP 581/591 C11 (L267, DOJINDO). Cellular and tissue samples were homogenized in RIPA lysis buffer (FD009; Fdbio), followed by centrifugation at 10000 × g for 10 min. An aliquot (0.1 mL of this supernatant) was combined with 0.2 mL of the MDA working solution in a centrifuge tube. This mixture was subjected to thermal treatment at 100 °C for 15 min and allowed to equilibrate to ambient temperature. The sample was centrifuged at 1000 × g for 10 min. A 200‐µL volume of the resulting supernatant was transferred to a 96‐well plate, and spectrophotometric analysis was performed at 532 nm using a microplate reader (Bio‐Rad Laboratories). For the fluorescent probe technique, HEI‐OC1 cells and basilar membranes were incubated with BDP working solution for 30 min.

### Ferrous Ion Detection

Ferro‐orange dye (F374, DOJINDO), mito‐ferrogreen dye (M489, DOJINDO), and an Iron Assay Kit (I291, DOJINDO) were used to detect ferrous ions. HEI‐OC1 cells and basilar membranes were exposed to 1 µmol L^−1^ Ferro‐Orange or 5 µmol L^−1^ Mito‐FerroGreen for 0.5 h in the cell incubator. The images were obtained after washing thrice with HBSS. HEI‐OC1 cells were evaluated using a BD Accuri C6 flow cytometer and the FlowJo X software to quantify the results. Each experiment was performed with five replicates.

### Immunofluorescence Staining

Mouse cochlear tissues and basilar membranes were initially fixed in 4% paraformaldehyde solution (1 h), followed by permeabilization with 0.1% TritonX‐100 (30 min). Immunolabeling was performed using primary antibodies (rabbit anti‐MyosinVIIa (25‐6790, Proteus BioSciences) and mouse anti‐SOCS1 (SC‐518028, Santa Cruz)) via overnight incubation at 4 °C after blocking using 10% goat serum (1 h, room temperature). The samples were incubated with corresponding secondary antibodies (1 h at room temperature).

### Regulation of SOCS1 In Vitro

HEI‐OC1 cells were transfected with 50 nmol/L siRNA‐SOCS1 using Lipo 3000 Transfection Reagent (L3000001; Invitrogen) for 24 h. The transfection efficiency of the knockdown was measured using real‐time Q‐PCR and WB. The siRNA‐SOCS1 sequence was as follows: sense: 5′‐UAACCCGGUACUCCGUGAC‐3, ’ antisense: 5′‐GUCACGGAGUACCGGGUUA‐3. ’ AAV viruses were used to regulate basilar membrane expression. The pAAV‐U6‐shRNA(SOCS1)‐CMV‐EGFP (1.51 × 1013 vg/mL) and pAAV‐CMV‐SOCS1‐T2A‐EGFP (1.24 × 1013 vg mL^−1^) vectors were purchased from Tsingke Biotechnology Co., Ltd. For the basilar membranes, the normal medium was replaced with an AAV‐containing medium at a concentration of 1 × 10^11^ g mL^−1^. After three days, the medium was replaced with normal medium and incubated for an additional two days, followed by subsequent procedures.

### RNA Extraction and Sequencing

Eight cultured basilar membranes from the different experimental groups were used for RNA extraction. The TRIzol reagent (TransGen, ET111‐01‐V2) was used to isolate total RNA. The extracted RNA was sent to Shanghai Personal Biotechnology Corporation for next‐generation sequencing. Genes were considered to be differentially expressed at q ≤ 0.05 and |log2_ratio| ≥ 1.

### Real‐Time Q‐PCR

One microgram of total RNA was used as the template for reverse transcription during the PCR amplification process. The relative expression levels of the genes were determined using the 2^−△△CT^ method. The primers used are listed in Table S1 (Supporting Information).

### Western Blotting and Co‐Immunoprecipitation Assay

After washing with phosphate‐buffered saline three times, HEI‐OC1 cells and cochlear tissues were lysed in RIPA buffer supplemented with a protease inhibitor cocktail (100×, FD1001, Fdbio) on ice for 15 min. The supernatants were denatured at 95 °C for 10 min and separated by 12% SDS‐PAGE (E301, Vazyme) after centrifugation. The protein bands were transferred to a polyvinylidene fluoride membrane after running the gels (IPVH00010, Millipore) and blocked with 5% defatted milk. The membrane was incubated with primary antibodies overnight at 4 °C. The antibodies were as follows: mouse anti‐SOCS1 (sc‐518028, Santa Cruz), rabbit anti‐FTH1 (A19544, ABclonal), rabbit anti‐SOCS1 (ab280886, Abcam), rabbit anti‐NCOA4 (A5695, ABclonal), mouse anti‐NCOA4 (sc‐373739, Santa Cruz), rabbit anti‐NCOA4 (YT0302, Immunoway), rabbit anti‐GPX4 (A11243, ABclonal), horseradish peroxidase‐labeled goat anti‐rabbit IgG (A0208, Beyotime), and goat anti‐mouse IgG (A0216, Beyotime). Mouse anti‐β‐Tubulin (AC021, ABclonal) and rabbit anti‐β‐Actin (AC038, ABclonal) were used as the internal reference proteins. The proteins were detected using a chemiluminescent substrate kit (34 580; Thermo Scientific) and analyzed using the ImageJ software.

### Auditory Brainstem Response

ABR recordings were conducted using the TDT system (Tucker Davis Technologies), as previously described.^[^ ^44^ ^]^ The mice were anesthetized with pentobarbital sodium at a dose of 100 mg kg^−1^ before the ABR measurements. The responses at each frequency level were analyzed, digitized, and averaged using BioSigRZ software.

### Distortion Product Otoacoustic Emission

An otoacoustic emission probe was inserted into the ear canal of each participant. Two primary tones (f1 and f2) were present in the probe. The cochlea responds to two primary tones by generating a third tone, known as the distortion product, at frequencies of 2f1–f2. The analyzer collected data on the amplitude and frequency of the DPOAEs. Measurements are typically performed at multiple frequencies (4, 8, 12, 16, 24, and 32 kHz) to assess the entire hearing frequency range.

### Endocochlear Potential

The animal was positioned in a stereotaxic frame to stabilize its head during the procedure. A small incision was made on the skin to expose the temporal bone. A portion of the temporal bone was carefully removed to expose the cochlea without damaging the surrounding structures. A glass microelectrode (5–8 MΩ) was used to penetrate the scala media. The electrode was filled with 150 mm of KCl to facilitate electrical measurements. The electrode should be positioned carefully to prevent damaging the delicate cochlear structures. The microelectrode was connected to an amplifier and data acquisition system (pClamp 10, Molecular Devices).

### Transmission Electron Microscopy

The HEI‐OC1 cells and the cochleae were harvested, prefixed with 3% glutaraldehyde (G1102, Servicebio) and post‐fixed in 1% OsO4 (18 456, Ted Pella Inc.). The cells were dehydrated, and the resin was penetrated and embedded. The embedding models were placed in a 60 °C oven to polymerize for over 48 h. The resin blocks were sectioned into 1.5 µm slices using a semi‐thin microtome (Leica UC7, Leica), followed by further cutting into 60–80 nm thin sections using an ultra‐microtome (Ultra 45°, Daitome). Staining was conducted and the subcellular structure was observed using a transmission electron microscope (HT7800, HITACHI).

### Statistical Analysis

Statistical analyses were performed using GraphPad Prism 8.0. Data are presented as the mean ± SD. All experiments were performed at least three times independently (*n* ≥ 3), and the mice in each group were more than three. Comparisons of the two groups were performed using two‐tailed unpaired Student's *t*‐tests. *p* < 0.05 denoted statistical significance.

## Conflict of Interest

The authors declare no competing interests.

## Supporting information



Supporting Information

## Data Availability

The data that support the findings of this study are available from the corresponding author upon reasonable request.
