# Supplementary material for: Pinoresinol Diglucoside Attenuates Nuclear Receptor Coactivator 4‐Mediated Ferritinophagy Associated with Cisplatin‐Induced Hearing Loss
Source: Adv Sci (Weinh). 2025 Mar 7;12(29):2408777. doi: 10.1002/advs.202408777 (PMC12362821; doi:10.1002/advs.202408777)
Supplement: Supplementary file 1 — Supporting Information [file ADVS-12-2408777-s001.docx]

Supporting Information 1

**Pinoresinol Diglucoside Attenuates Nuclear Receptor Coactivator 4-Mediated Ferritinophagy Associated With Cisplatin-Induced Hearing Loss**

*Yin Chen^1,2^, Cheng Cheng^1,2^, Ao Li^1,2^, Dengbin Ma^1,2^, Siyu Li^1,2^, Handong Wang^1,2^, Song Gao^1,2^, Dingding Liu^1,2^, Panpan Song^1,2^, Chenjie Yu*^1, 2^, Xiaoyun Qian*^1, 2^, Guoqiang Wan*^1, 2 ,3^, Xia Gao*^1, 2^*


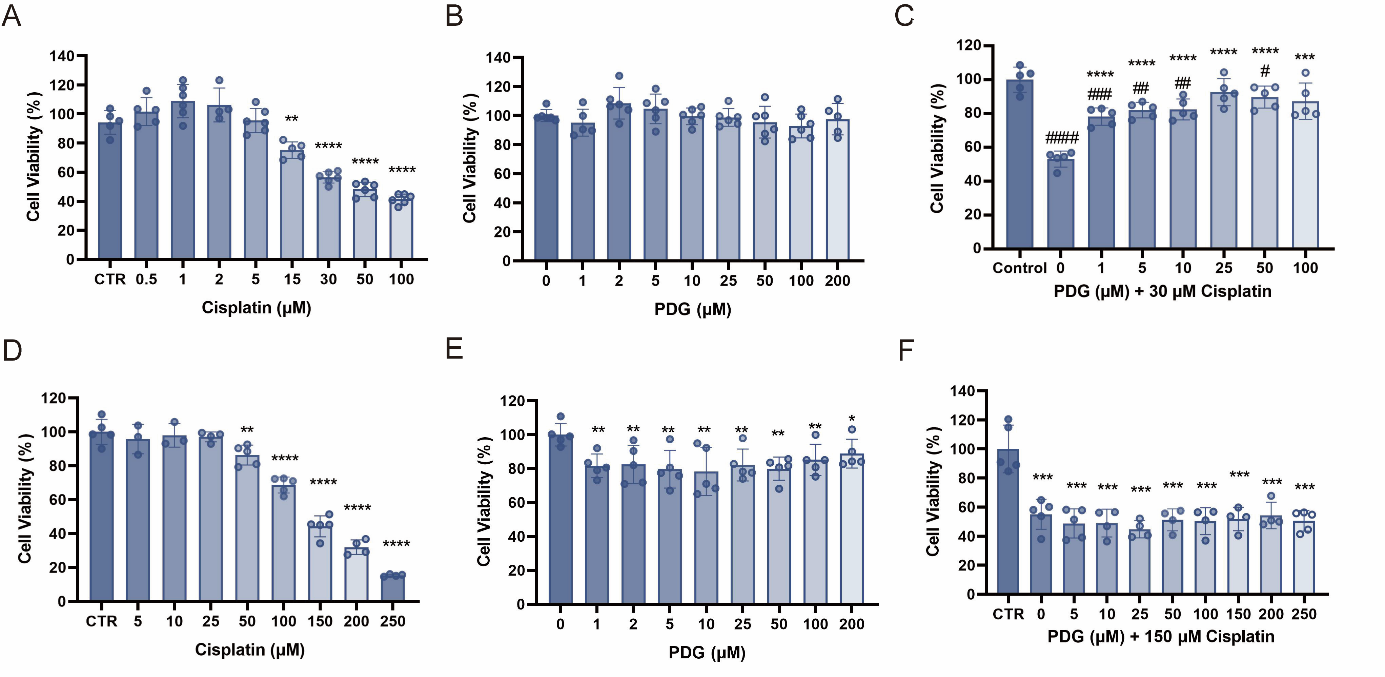


**Figure S1.** Effects of PDG on cells. (A) Cell viability in different groups treated with different concentration of cisplatin in HEI-OC1 cells measured with CCK-8 to determine the optimal drug concentration; n = 5. (B) Cell viability in different groups treated with different concentration of PDG in HEI-OC1 cells measured with CCK-8; n = 5. (C) Cell viability in different treatment groups measured with CCK-8 to determine the optimal drug concentration; n = 5. (D) Cell viability in different groups treated with different concentrations of cisplatin in Cal-27 cells measured with CCK-8 to determine the optimal drug concentration; n = 5. (E) Cell viability in different groups treated with different concentration of PDG in Cal-27 cells measured with CCK-8; n = 5. (F) Cell viability in different treatment groups measured with CCK-8 in Cal-27 cells. n = 5. The data are presented as mean ± S.D. * p < 0.05, ** p < 0.01, *** p < 0.001, **** p < 0.0001, n.s. = not significant versus the Cisplatin group and ##p < 0.01 and ###p < 0.001 versus the Control group, two-tailed, unpaired Student’s t-tests.


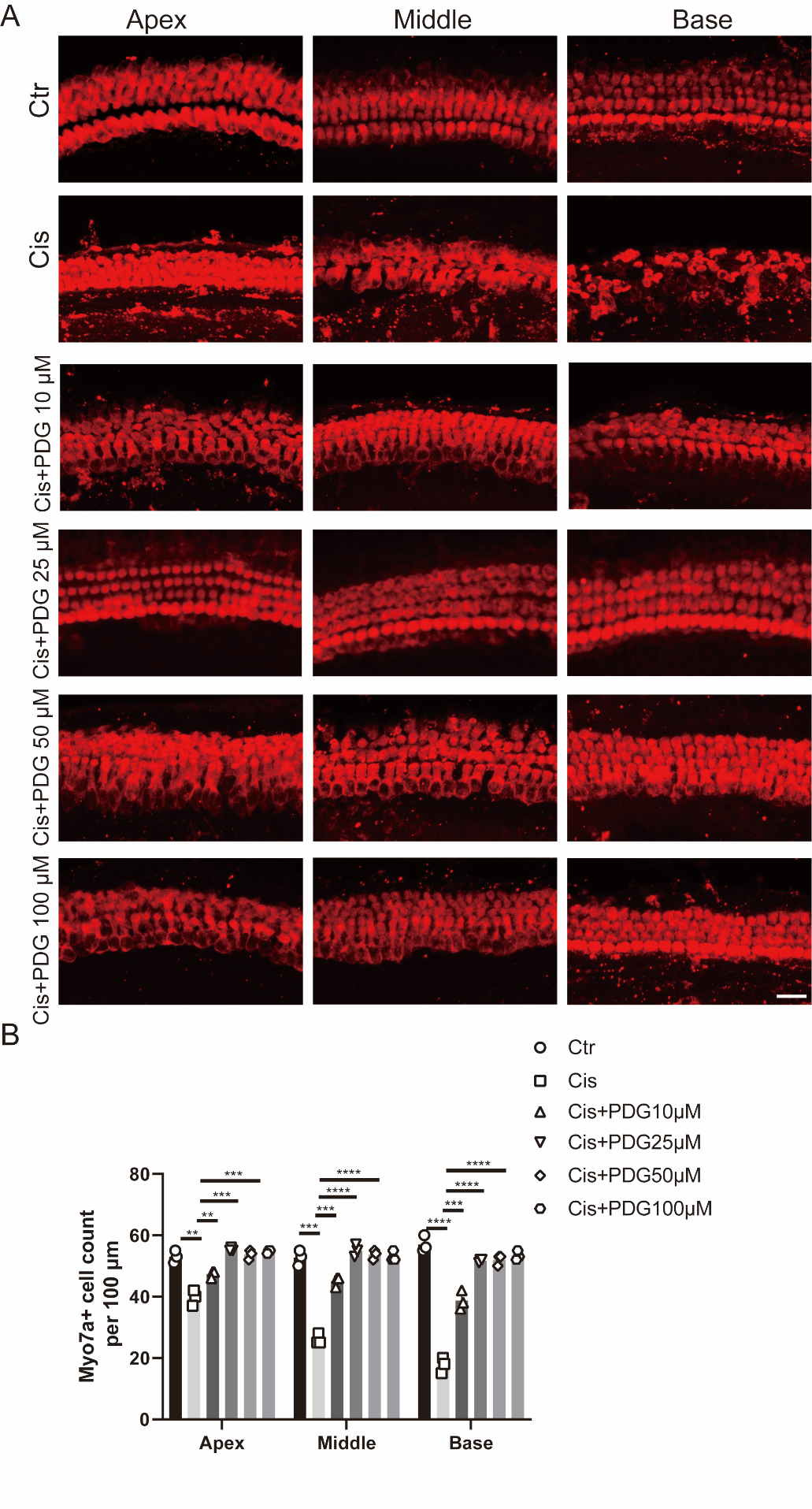


**Figure S2.** Effects of PDG on cochlear HCs. (A) Immunostaining of MyosinVIIa (red) in basilar membranes from various treatment groups to determine the optimal drug concentration; n = 3. (B) Quantitation of MyosinVIIa-positive HCs in the base, middle, and apex of basilar membranes in the different groups; n = 3; The data are presented as the mean ± S.D. * p < 0.05, ** p < 0.01, *** p < 0.001, **** p < 0.0001, n.s. = not significant, two-tailed, unpaired Student’s t-tests.
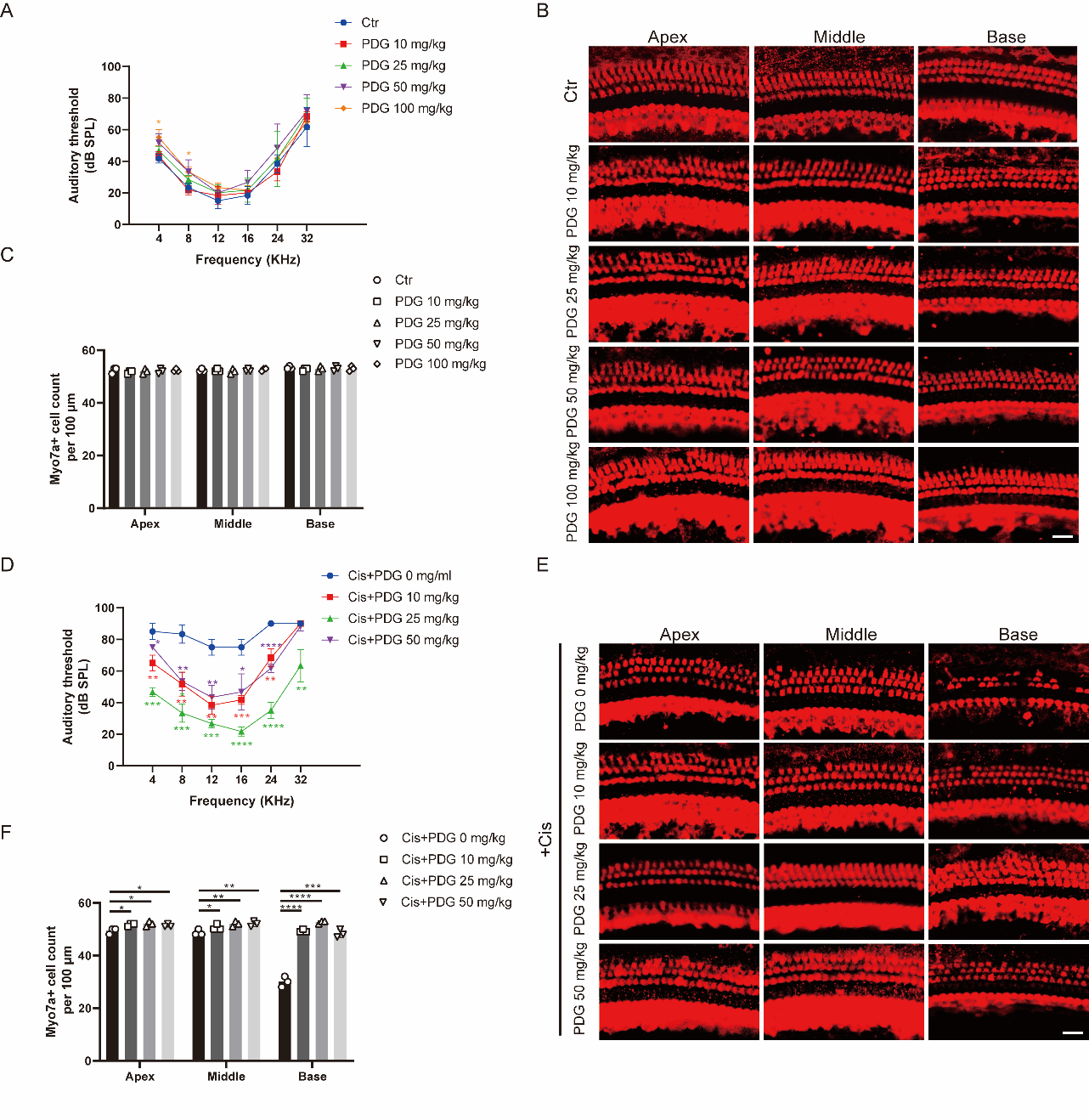


**Figure S3.** Effects of PDG in vivo. (A) ABR thresholds and (B) Immunostaining of MyosinVIIa (red) in HCs from various treatment groups to determine the optimal drug concentration; n = 3. (C) Quantitation of MyosinVIIa-positive HCs in the base, middle, and apex of basilar membranes in the different groups; n = 3. (D) ABR thresholds and (E) Immunostaining of MyosinVIIa (red) in HCs from various treatment groups to determine the optimal drug concentration against cisplatin; n = 3. (F) Quantitation of MyosinVIIa-positive HCs in the base, middle, and apex of basilar membranes in the different groups; n = 3. The data are presented as the mean ± S.D. * p < 0.05, ** p < 0.01, *** p < 0.001, **** p < 0.0001, n.s. = not significant, two-tailed, unpaired Student’s t-tests.


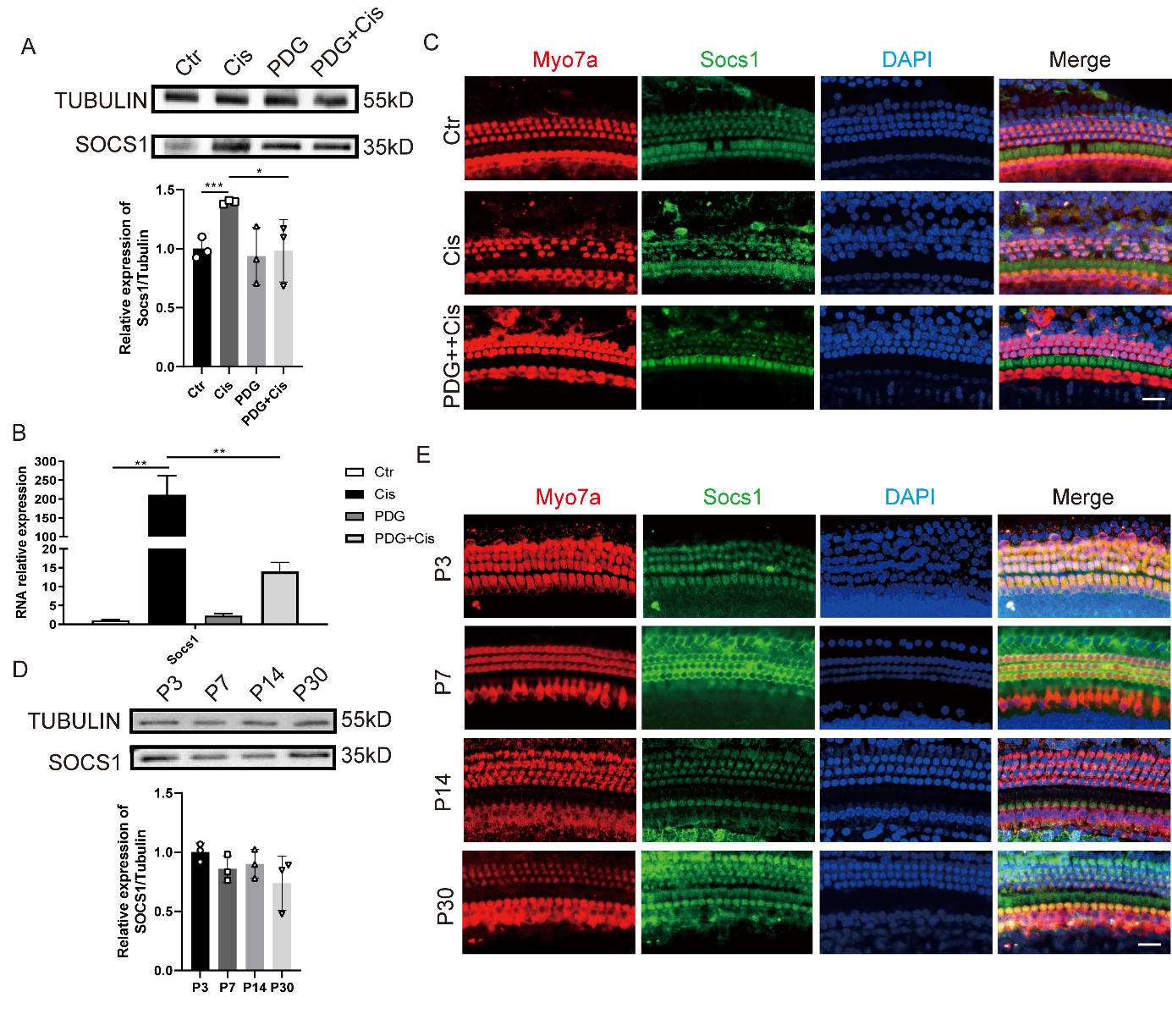


**Figure S4.** The expression of Socs1 in cochlea. (A)(B) Relative expression levels of SOCS1 by WB and Q-PCR after different treatment in vivo; n = 3. (C) Immunofluorescence staining for MyosinVIIa (red), SOCS1 (green), and DAPI (blue) showing SOCS1 expression in HCs after different treatment in vivo; n = 3. Scale bar: 20 μm. (D) Relative expression levels of SOCS1 by WB analysis at four different ages (P3, P7, P14, P30); n = 3. (E) Immunofluorescence staining for MyosinVIIa (red), SOCS1 (green), and DAPI (blue) showing SOCS1 expression in HCs at different ages; n = 3. Scale bar: 20 μm. The data are presented as the mean ± S.D. * p < 0.05, ** p < 0.01, *** p < 0.001, **** p < 0.0001, n.s. = not significant, two-tailed, unpaired Student’s t-tests.


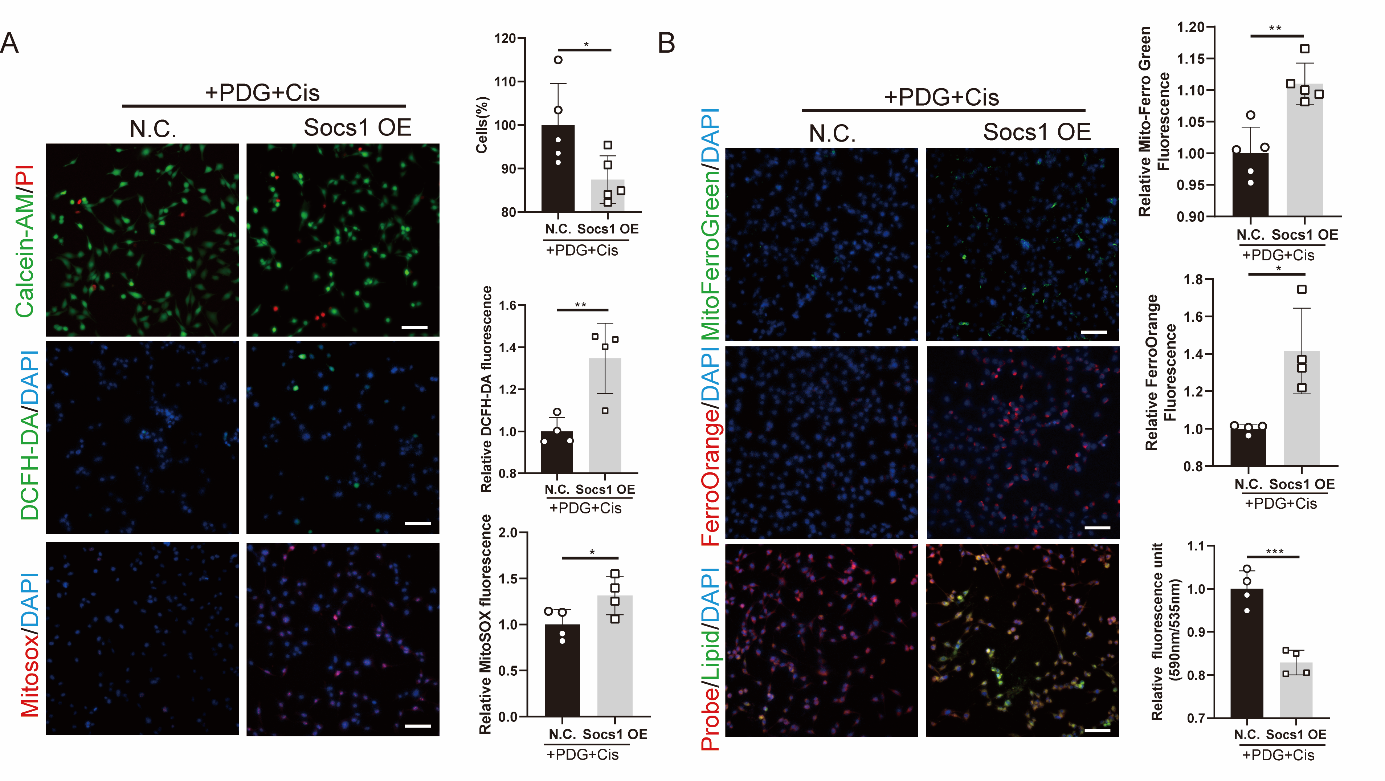


**Figure S5.** Overexpression of Socs1 aggravates cisplatin-induced oxidative stress and ferroptosis in HEI-OC1 cells. (A) Overexpression of Socs1 aggravates cisplatin-induced oxidative stress in HEI-OC1 cells. Live /dead, DCFH-DA and mitoSOX staining images and quantitative analysis of the relative fluorescence intensity of HEI-OC1 cells incubated under different treatments; n = 5. Scale bar: 100 μm. (B) Overexpression of Socs1 aggravates cisplatin-induced ferroptosis in HEI-OC1 cells. Mito-FerroGreen, Ferro-Orange and BDP staining images and quantitative analysis of the relative fluorescence intensity of HEI-OC1 cells incubated under different treatments; n = 5. Scale bar: 100 μm. The data are presented as the mean ± S.D. * p < 0.05, ** p < 0.01, *** p < 0.001, **** p < 0.0001, n.s. = not significant, two-tailed, unpaired Student’s t-tests.


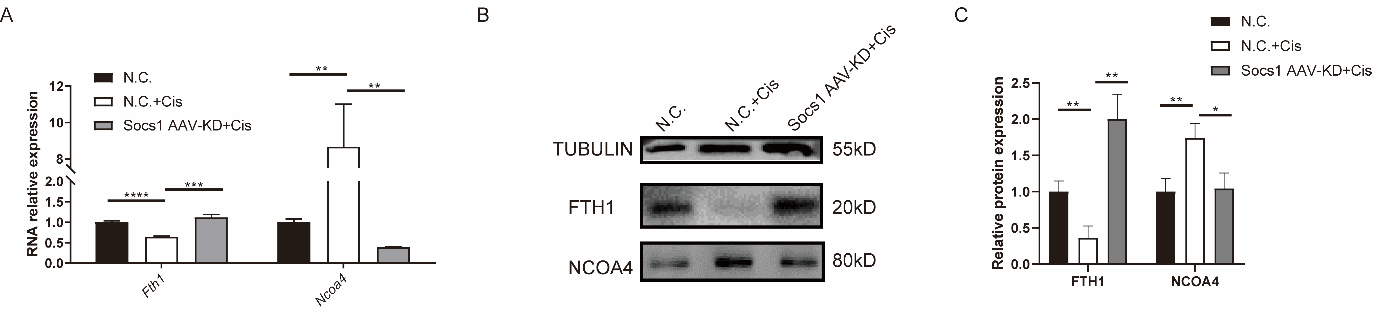


**Figure S6.** Downregulating *Socs1*alleviated NCOA4-induced ferritinophagy in ex vivo cultures. (A) Q-PCR analysis of ferroautophagy-related genes in basilar membranes with SOCS1 knockdown; n = 4. (B-C) WB analysis of FTH1 and NCOA4 in basilar membranes with SOCS1 knockdown; n = 3. The data are presented as the mean ± S.D. * p < 0.05, ** p < 0.01, *** p < 0.001, **** p < 0.0001, n.s. = not significant, two-tailed, unpaired Student’s t-tests.


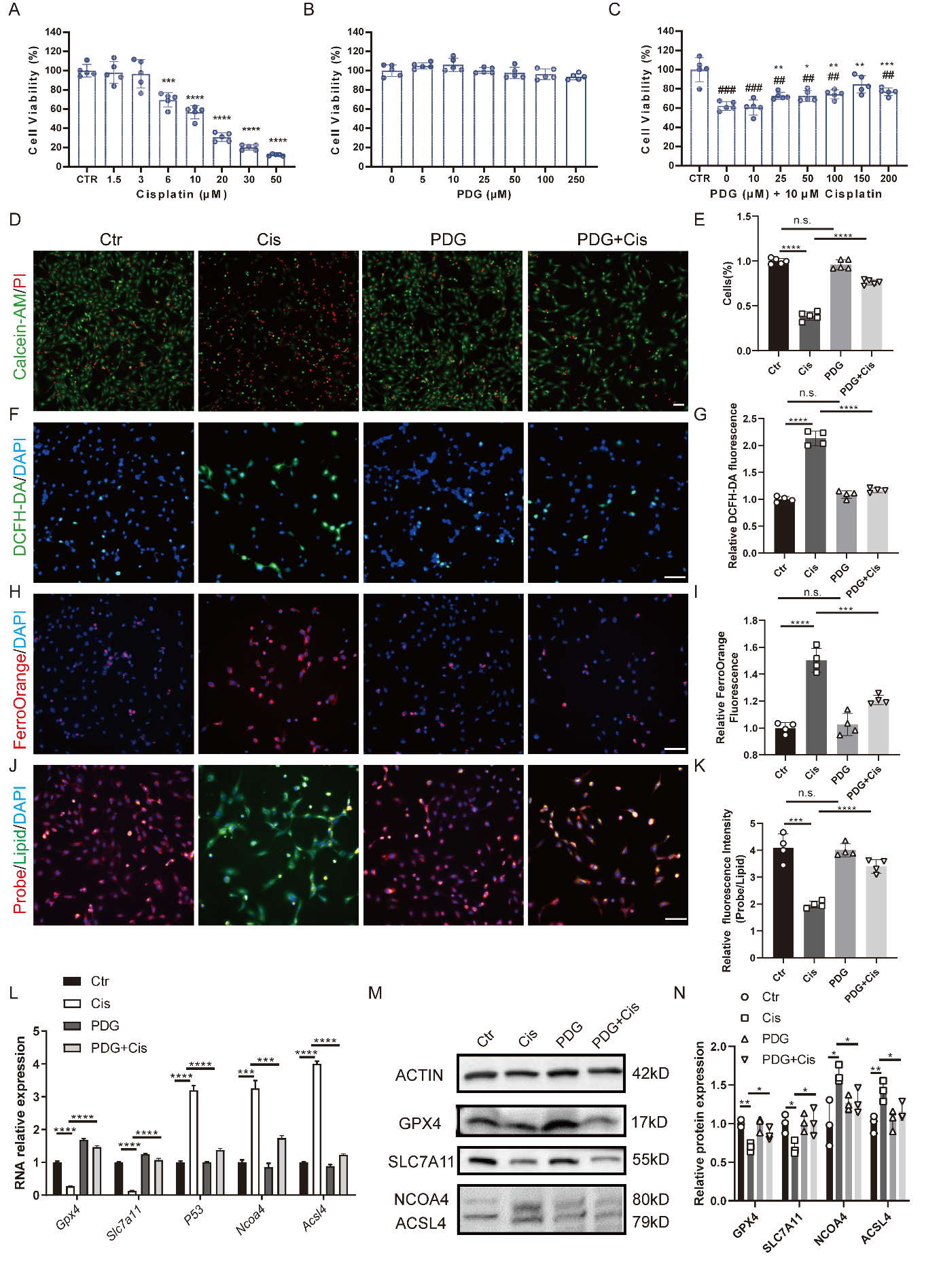


**Figure S7.** Effects of PDG in SGN cells. (A) Cell viability in different groups treated with different concentration of cisplatin in SGN cells measured with CCK-8 to determine the optimal drug concentration; n = 5. (B) Cell viability in different groups treated with different concentration of PDG in SGN cells measured with CCK-8; n = 5. (C) Cell viability in different treatment groups measured with CCK-8 to determine the optimal drug concentration; n = 5. (D-G) PDG alleviated cisplatin-induced oxidative stress in HEI-OC1 cells. Live /dead and DCFH-DA staining images and quantitative analysis of the relative fluorescence intensity of SGN cells incubated under different treatments; n = 4. Scale bar: 100 μm. (H-K) PDG alleviated cisplatin-induced ferroptosis in SGN cells. Ferro-Orange and BSP staining images and quantitative analysis of the relative fluorescence intensity of SGN cells incubated under different treatments; n = 4. Scale bar: 100 μm. (L) Q-PCR analysis of ferrptosis-related genes in SGN cells; n = 4. (M-N) WB analysis of ferrptosis-related protein in SGN cells with different treatments; n = 3. The data are presented as the mean ± S.D. * p < 0.05, ** p < 0.01, *** p < 0.001, **** p < 0.0001, n.s. = not significant, two-tailed, unpaired Student’s t-tests.

**Table S1.** The primers of different genes.

| Gene | Forward Sequence | Reverse Sequence |
| --- | --- | --- |
| H2ac18 | TCAGCACTGCGTCTGCACC | ATGCGCGTCTTCTTGTTGTC |
| Scand1 | CGCAGAGAAGCCAGAGACTT | TCAGCACTGCGTCTGCACC |
| H2aj | ACAACAAGAAGACGCGCATC | TCACTTGCTCTTCACCTTCTG |
| Jund | GAAACGCCCTTCTATGGCGA | CAGCGCGTCTTTCTTCAGC |
| Socs1 | CTGCGGCTTCTATTGGGGAC | AAAAGGCAGTCGAAGGTCTCG |
| Cebpd | CGACTTCAGCGCCTACATTGA | CTAGCGACAGACCCCACAC |
| Gpx4 | GCCTGGATAAGTACAGGGGTT | CATGCAGATCGACTAGCTGAG |
| Slc7a11 | CTTTGTTGCCCTCTCCTGCTTC | CAGAGGAGTGTGCTTGTGGACA |
| Fth1 | CAAGTGCGCCAGAACTACCA | GCCACATCATCTCGGTCAAAA |
| Ftl | CGCTCAAAGAGATACTCGCC | CGCTCAAAGAGATACTCGCC |
| Ncoa4 | GAACCATCAGGACACATGGAAA | AGGAGCCATAGCCTTGGGT |
| Actin | GGCTGTATTCCCCTCCATCG | GGCTGTATTCCCCTCCATCG |

Supporting Information 2

**Pinoresinol Diglucoside Attenuates Nuclear Receptor Coactivator 4-Mediated Ferritinophagy Associated With Cisplatin-Induced Hearing Loss**

*Yin Chen^1,2^, Cheng Cheng^1,2^, Ao Li^1,2^, Dengbin Ma^1,2^, Siyu Li^1,2^, Handong Wang^1,2^, Song Gao^1,2^, Dingding Liu^1,2^, Panpan Song^1,2^, Chenjie Yu*^1, 2^, Xiaoyun Qian*^1, 2^, Guoqiang Wan*^1, 2 ,3^, Xia Gao*^1, 2^*

Original images of gel blots

Supporting Data 1: This images represent the original data used to generate the results presented in Figure 4H.

PDG+Cis


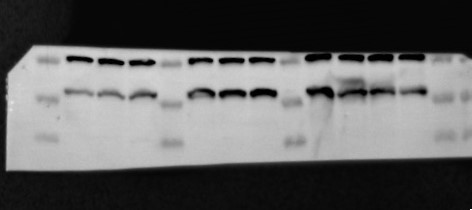


ACTIN 42kda

Ctr

Cis


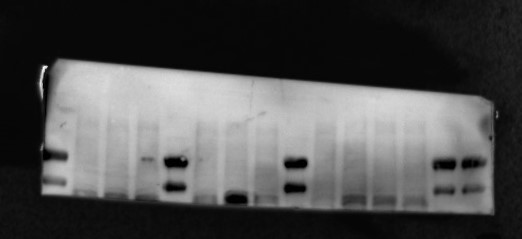

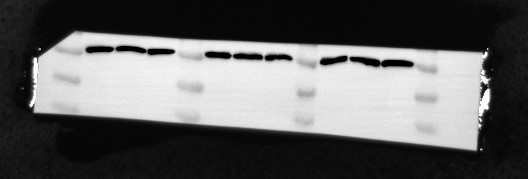

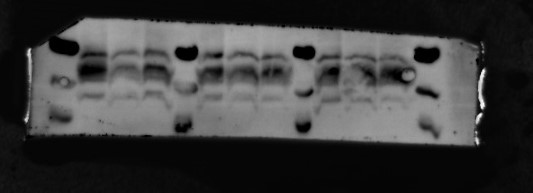

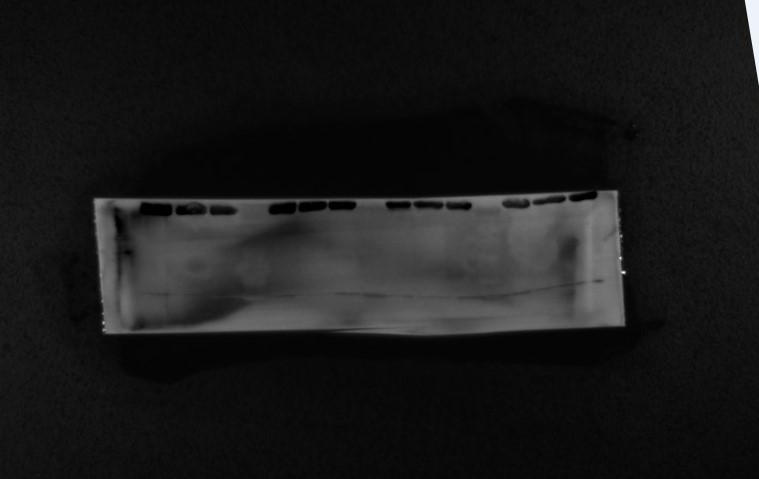

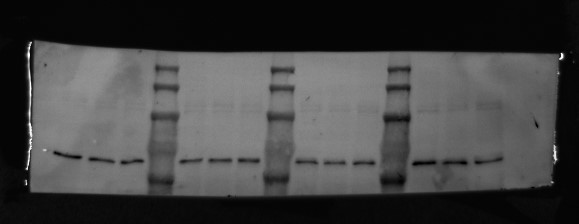


PDG+Cis

Cis

Ctr

PDG+Cis

Cis

Ctr

ACTIN 42kda

NCOA4 80kda

Ctr

Cis

PDG+Cis

GPX4 17kda

Cis

Ctr

PDG+Cis

ACTIN 42kda

Ctr

PDG+Cis

Cis

SLC7A11 55kda

Supporting Data 2: This images represent the original data used to generate the results presented in Figure 5D.


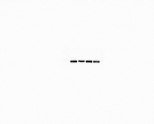

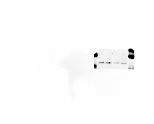


PDG+Cis

PDG

Cis

Ctr

SOCS1 35kda

PDG

Cis

Ctr

PDG+Cis

TUBULIN 55kda

Supporting Data 3: This images represent the original data used to generate the results presented in Figure 5F.


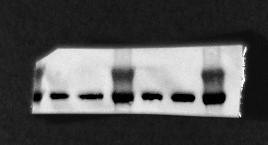

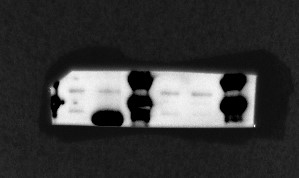


N.C.

N.C.

Socs1-AAV-OE

Socs1-AAV-OE

SOCS1 35kda

TUBULIN 55kda

Supporting Data 4: This images represent the original data used to generate the results presented in Figure 6A.


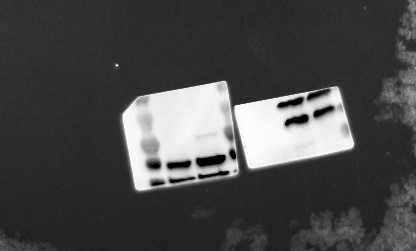

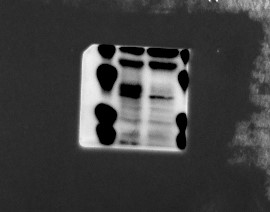


Socs1-AAV-KD

N.C.

N.C.

Socs1-AAV-KD

SOCS1 35kda

TUBULIN 55kda

Supporting Data 5: This images represent the original data used to generate the results presented in Figure 7A.


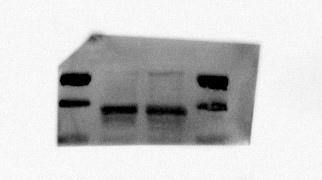

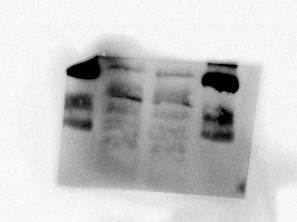


N.C.

Socs1-KD

Socs1-KD

N.C.

SOCS1 35kda

TUBULIN 55kda

Supporting Data 6: This images represent the original data used to generate the results presented in Figure 7Q.

N.C.+Cis

Socs1 KD+Cis

N.C.


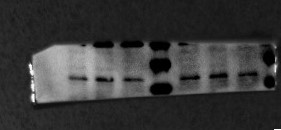

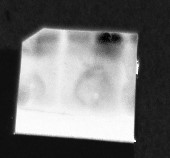

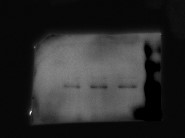

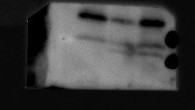


GPX4 17kda

FTH1 20kda

Socs1 KD+Cis

N.C.

N.C.+Cis

Socs1 KD+Cis

N.C.+Cis

N.C.

Socs1 KD+Cis

N.C.+Cis

N.C.

NCOA4 80kda

TUBULIN 55kda

Supporting Data 6: This images represent the original data used to generate the results presented in Figure 7S.


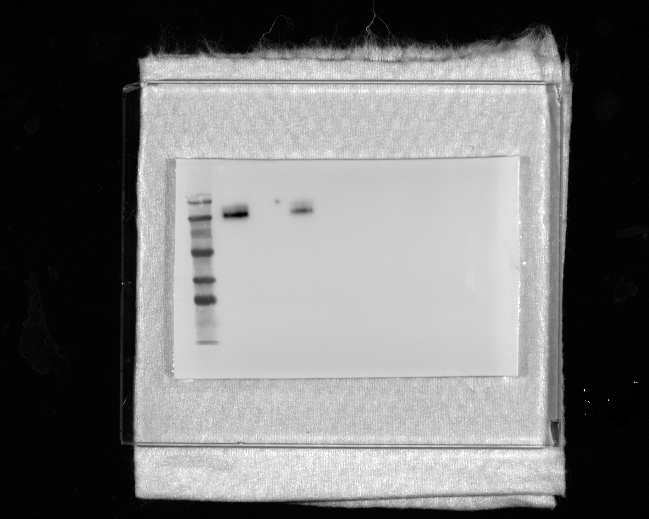

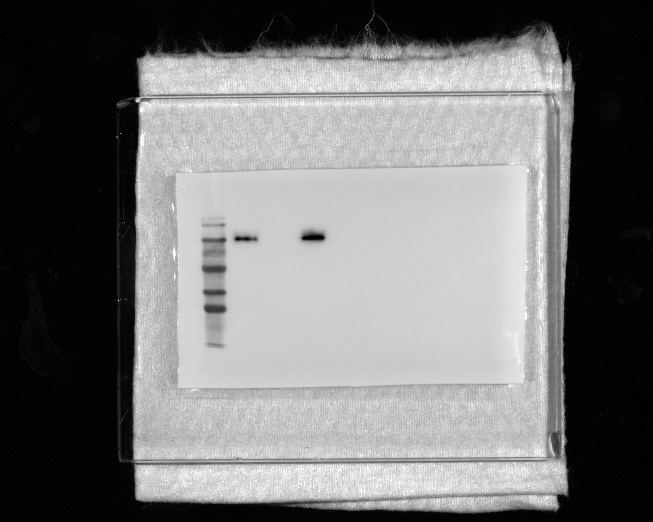

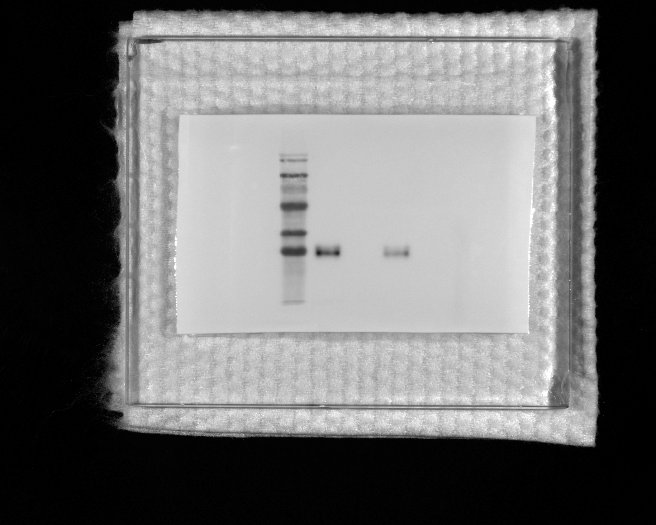

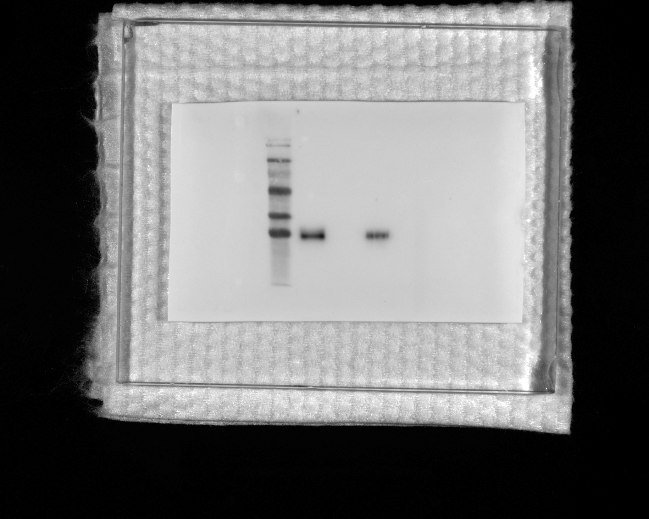


Socs1 35kda

(IB)

Socs1 35kda

(IP)

NCOA4 70kda

(IP)

NCOA4 70kda

(IB)

Supporting Data 7: This images represent the original data used to generate the results presented in Figure S4A.

PDG+Cis

Ctr

Cis

PDG

PDG+Cis


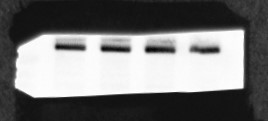

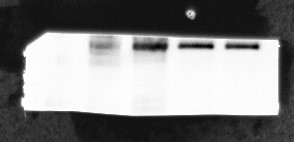


Ctr

Cis

PDG

TUBULIN 55kda

SOCS1 35kda

Supporting Data 8: This images represent the original data used to generate the results presented in Figure S4D.


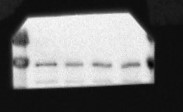

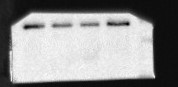


P3

P7

P30

P14

P30

P14

P7

P3

SOCS1 35kda

TUBULIN 55kda

Supporting Data 9: This images represent the original data used to generate the results presented in Figure S6B.


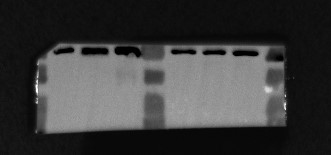

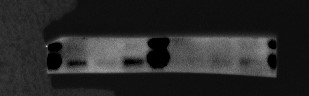

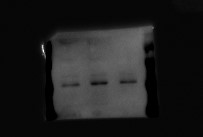


TUBULIN 55kda

FTH1 20kda

NCOA4 80kda

N.C.

N.C.+Cis

Socs1 AAV KD+Cis

Socs1 AAV KD+Cis

N.C.+Cis

N.C.

Socs1 AAV KD+Cis

N.C.+Cis

N.C.

Supporting Data 10: This images represent the original data used to generate the results presented in Figure S7M.


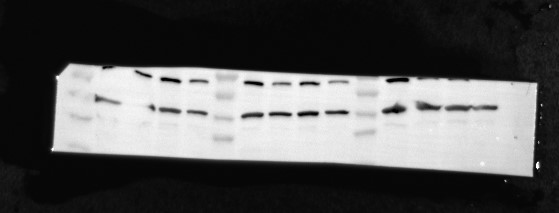


ACTIN 42kda

SLC7A11 55kda

PDG+Cis

PDG

Cis

Ctr


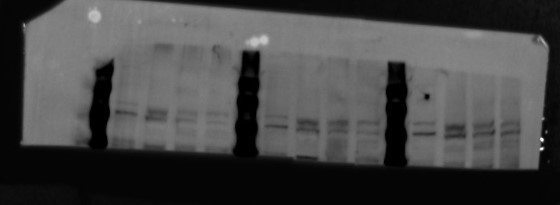

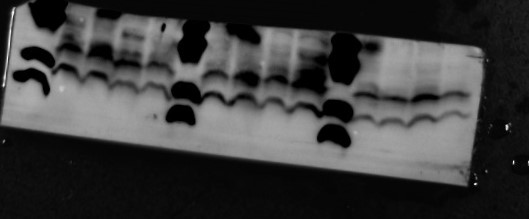


ACSL4 79kda

NCOA4 80kda

Ctr

Cis

PDG

PDG+Cis

Cis

Ctr

PDG

PDG+Cis

GPX4 17kda


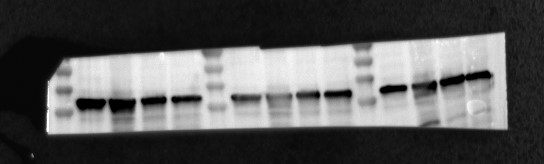


ACTIN 42kda

Cis

Ctr

PDG+Cis

PDG

Supporting Information 3

We use the resource equation to calculate the sample size required for animal experiments. The calculation formula of the resource equation is as follows:

E=N-K=Kn-K=K(n-1)

Where N is the total number of experimental units, n is the sample size of each group, K is the number of treatment groups, and E is the difference between the two. The above formula can be converted as follows: n = E/K+1. The minimum value of E is 10 and the maximum value is 20. Thus the minimum and maximum number of animals in each group can be obtained: Min n = 10 / K+1, Max n = 20 / K+1.

In our in vivo experiments, mice were divided into four groups, so Min n =10/4+1=3.5, Max n =20/4+1=6.

Therefore, the number of animals per experiment per group should be 4-6. Thus n=5 for the ABR experiment and n=5 for the DPOAE experiment was appropriate. A total of n = 10 mice per group was decided. After completing the above ABR and DPOAE experiments to prove the protective effect of PDG on the hearing of mice, we used 10 mice respectively for subsequent immunofluorescence, WB, Q-PCR and transmission electron microscopy experiments.

Eight-week-old male C57BL/6 mice (purchased from Huachuang Sino) were used in this study. All mice were housed under controlled environmental conditions (temperature: 22 ± 2°C; humidity: 50 ± 10%; 12-hour light/dark cycle) and were allowed to acclimate for at least 7 days before the start of the experiment. Mice were randomly assigned to four experimental groups: control, cisplatin, PDG, and PDG + cisplatin (n = 10 per group). Randomization was performed using a computer-generated random sequence to avoid bias.

Cisplatin was prepared as a 0.5 mg/ml solution with sterile normal saline, and PDG was prepared as a 2.5 mg/ml solution in 10% DMSO, 40% PEG300, and 5% Tween-80.

Mice in the cisplatin group were administered cisplatin (4 mg/kg, intraperitoneal injection) daily for 4 consecutive days, followed by a 10-day recovery period with no other interventions. After the recovery, cisplatin injections were repeated for 4 days, followed by another 10-day recovery period. This cycle of 4 days of cisplatin injection and 10 days of recovery was repeated until the sample was collected on day 42. Mice in the PDG + cisplatin group received concurrent intraperitoneal injections of PDG (25 mg/kg) and cisplatin (4 mg/kg) at the same time for 42 days (4-day injection followed by 10-day recovery, repeated three times). Mice in the control and PDG groups received saline or PDG (25 mg/kg, intraperitoneal injection) for 42 days (4-day injection followed by 10-day recovery, repeated three times). All injections were performed using volumes not exceeding 200 µL per mouse.

All experimental procedures were in compliance with ethical guidelines for laboratory animal use and were approved by the Institutional Animal Care and Use Committee of Nanjing University (Approval No. 2021AE01090). The study protocol adhered to the ARRIVE 2.0 guidelines to ensure transparency, reproducibility, and ethical conduct. To ensure consistency, the health and welfare of the animals were monitored throughout the study. All mice had free access to standard rodent chow and tap water ad libitum. The experimenters were blinded to the group allocation during the assessment phase to reduce potential biases in outcome measurement.
